# Robust Rapid Cellular Metabolite Sensing Using Benchtop NMR and SABRE-Hyperpolarized [1-^13^C]Pyruvate

**DOI:** 10.1021/acs.analchem.5c05076

**Published:** 2026-03-10

**Authors:** Joseph Gyesi, Patrick TomHon, Abubakar Abdurraheem, Anna Samoilenko, Sydney Scofield, Clementinah Oladun, Stephen McBride, Erica Curran, Megan Pike, Kamal Kadari, Sydney D. Welch, Sam Lipka, Steven Balboa, Charlie Fehl, Marianna Sadagurski, Jan-Bernd Hövener, Thomas Theis, Boyd M. Goodson, Eduard Y. Chekmenev

**Affiliations:** † Department of Chemistry, Integrative Biosciences (Ibio), Karmanos Cancer Institute (KCI), 189376 Wayne State University, Detroit, Michigan 48202, United States; ‡ Biological Sciences, Institute of Environmental Health Sciences, Wayne State University, Detroit, Michigan 48202, United States; § Department of Chemistry, 6798 NC State University, Raleigh, North Carolina 27606, United States; ∥ Section Biomedical Imaging, Molecular Imaging North Competence Center (MOIN CC) Department of Radiology and Neuroradiology, University Medical Center Kiel, Kiel University, Am Botanischen Garten 18, Kiel 24118, Germany; ⊥ Comparative Medicine Institute, NC State University, Raleigh, North Carolina 27606, United States; # School of Chemical & Biomolecular Sciences, 2254 Southern Illinois University, Carbondale, Illinois 62901, United States

## Abstract

Hyperpolarized NMR has emerged as a powerful analytical technique to significantly enhance targeted NMR signals, improving the sensitivity for investigations of unique chemical and biological dynamics. Here, we demonstrate the use of a hyperpolarization strategy based on Signal Amplification By Reversible Exchange (SABRE) to generate highly reproducible doses of a hyperpolarized [1-^13^C]­pyruvate probe for benchtop characterization of yeast metabolism. This method allows rapid, scalable, and benchtop preparation of biocompatible hyperpolarized solutions suitable for live-cell experiments. We show that this production can be dove-tailed into a modular, compact workflow to characterize real-time metabolism in cell cultures, using *Saccharomyces cerevisiae* (Baker’s yeast) as a model organism. With high temporal resolution, we show that this method can resolve the conversion of hyperpolarized [1-^13^C]­pyruvate into oxidative decarboxylation products CO_2_ and bicarbonate. This conversion exhibits sustained and detectable metabolic activity for over 300 s after introduction of the agent to the cells. We model the metabolite kinetics to show decarboxylation activity and derive estimates of the pH over time from the CO_2_ and bicarbonate (carbonic acid buffer system) equilibrium to probe changes in the cellular environment during active metabolism. These results highlight the utility of benchtop SABRE-hyperpolarized [1-^13^C]­pyruvate as a scalable, specific probe for metabolic phenotyping of living cells using compact, low-cost instrumentation well-suited for future high-throughput applications across microbial engineering, drug response profiling, and dynamic metabolic screening.

## Graphical abstract

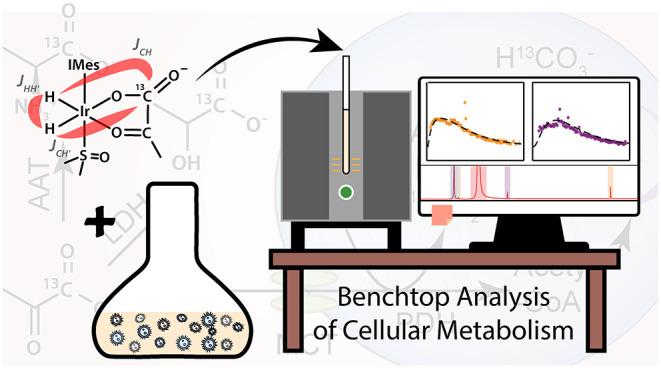

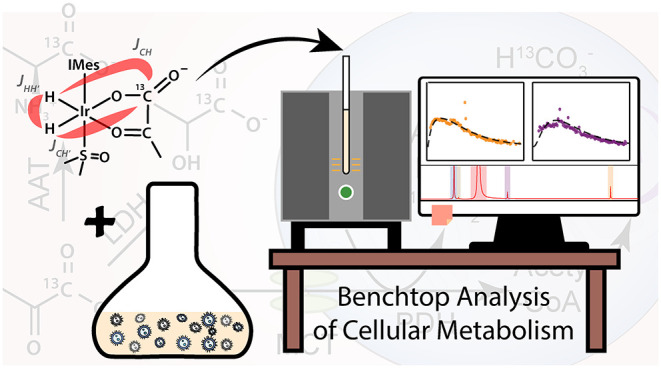

## Introduction

High-throughput metabolic analysis is central to many areas of biological research, from cancer biology to microbial engineering.^1–3^ Yet, despite advances in the engineering of systems biology and advances in cellular modeling, most *in vitro* metabolic assays remain limited by trade-offs between sensitivity, specificity, and scalability. High-accuracy quantitative mass spectrometry of extracted metabolites can provide detailed end point information, but typically requires destructive sampling, label incorporation, or complex sample preparation.^4,5^ In contrast, optical methods such as fluorescence-based optical readouts offer high sensitivity and compatibility with live-cell, nonterminal imaging, but their limited chemical specificity, quantitative variability, and reliance on exogenous probes or genetic modification can complicate interpretation and perturb native biology.^6,7^ As a result, real-time, quantitative, nonterminal tracking of endogenous metabolites across large sample sets remains a significant unmet need. This gap limits the pace of discovery in fields in which temporal resolution and quantitative insight into central metabolism are critical. Additionally, to align with NIH-supported efforts to reduce reliance on animal models through new approach methodologies (NAMs) there is increasing demand for analytical tools that enable detailed, mechanistic studies in nonanimal systems.^8,9^ Applications ranging from drug screening in mammalian cells to pathway optimization in engineered microbes would benefit from a broadly accessible, high-throughput tool capable of monitoring native metabolic conversion in real time with high sensitivity.

Hyperpolarized (HP) magnetic resonance has emerged as a transformative technique to address this challenge, offering NMR signal enhancements of 10,000-fold or more for select molecular species.^10–12^ Specifically, ^13^C-labeled metabolic substrates such as pyruvate can be easily hyperpolarized and have high sensitivity to metabolic dynamics, revealing key quantitative and qualitative insights into subject biology, such as enzymatic flux through glycolytic and oxidative pathways.^13–15^ Among HP methods, dissolution dynamic nuclear polarization (d-DNP) remains the gold standard, offering high polarization levels and compatibility with a range of molecular targets. d-DNP has enabled clinical translation of HP [1-^13^C]­pyruvate for *in vitro* studies of cell metabolism,^16–19^ *in vivo* imaging of cardiological, oncological, and neurological diseases in preclinical animal models,^20–24^ and even *in vivo* visualization and quantification of cancer biology in human patients.^14,25–27^ Despite the success with d-DNP technology, the infrastructure requirements, including cryogenic conditions and high cost of installation, can limit widespread technology adoption and constrain access for some laboratories.^12,27^

Parahydrogen-based methods, including hydrogenative parahydrogen-induced polarization (such as side arm hydrogenative, SAH-PHIP), offer a faster and more generalized lab-scale alternative to d-DNP.^28,29^ SAH-PHIP enables rapid polarization by chemically incorporating a pure spin isomer of parahydrogen gas into molecular precursors, followed by a chemical transformation (e.g., hydrolysis) to release the HP target. Although this approach has achieved suitable polarization levels for a breadth of applications including *in vivo* imaging and has demonstrated robust metabolic tracking and imaging of pyruvate and its downstream metabolites such as lactate,^29,30^ it requires precursor synthesis and side arm cleavage.^29^

SABRE (Signal Amplification By Reversible Exchange) is a complementary parahydrogen-based approach that circumvents many inherent limitations of SAH-PHIP by transferring spin order directly from parahydrogen to the target molecule via transient binding to a metal complex (typically an iridium N-heterocyclic carbene complex, Figure 1C), without the need for permanent chemical transformation.^31–34^ As a result, SABRE can avoid the precursor synthesis and the side arm cleavage steps. Some SABRE demonstrations have shown that near unity polarization is possible^35^ and the method offers several advantages that make it uniquely amenable to benchtop and high-throughput workflows. These include rapid polarization cycles (on the order of tens of seconds), the ability to maintain a repolarizable bolus of HP solution, and straightforward preparation of the HP reagent using inexpensive hardware and room-temperature solvents.^32,36,37^ The applicability of SABRE has been enhanced by robust extensions of the core method to long-lived heteronuclei (SABRE in SHIELD Enables Alignment Transfer to Heteronuclei, SABRE-SHEATH)^34,38,39^ and introduction of coligands for the extension of the method to key metabolites such as pyruvate.^34,40,41^ Furthermore, recent advances, notably the development of biocompatible solvent systems like Ace-SABRE and modular purification strategies, have enabled the direct generation of injectable, biocompatible HP solutions of metabolites such as [1-^13^C]­pyruvate.^32,42–44^

**Figure 1:**
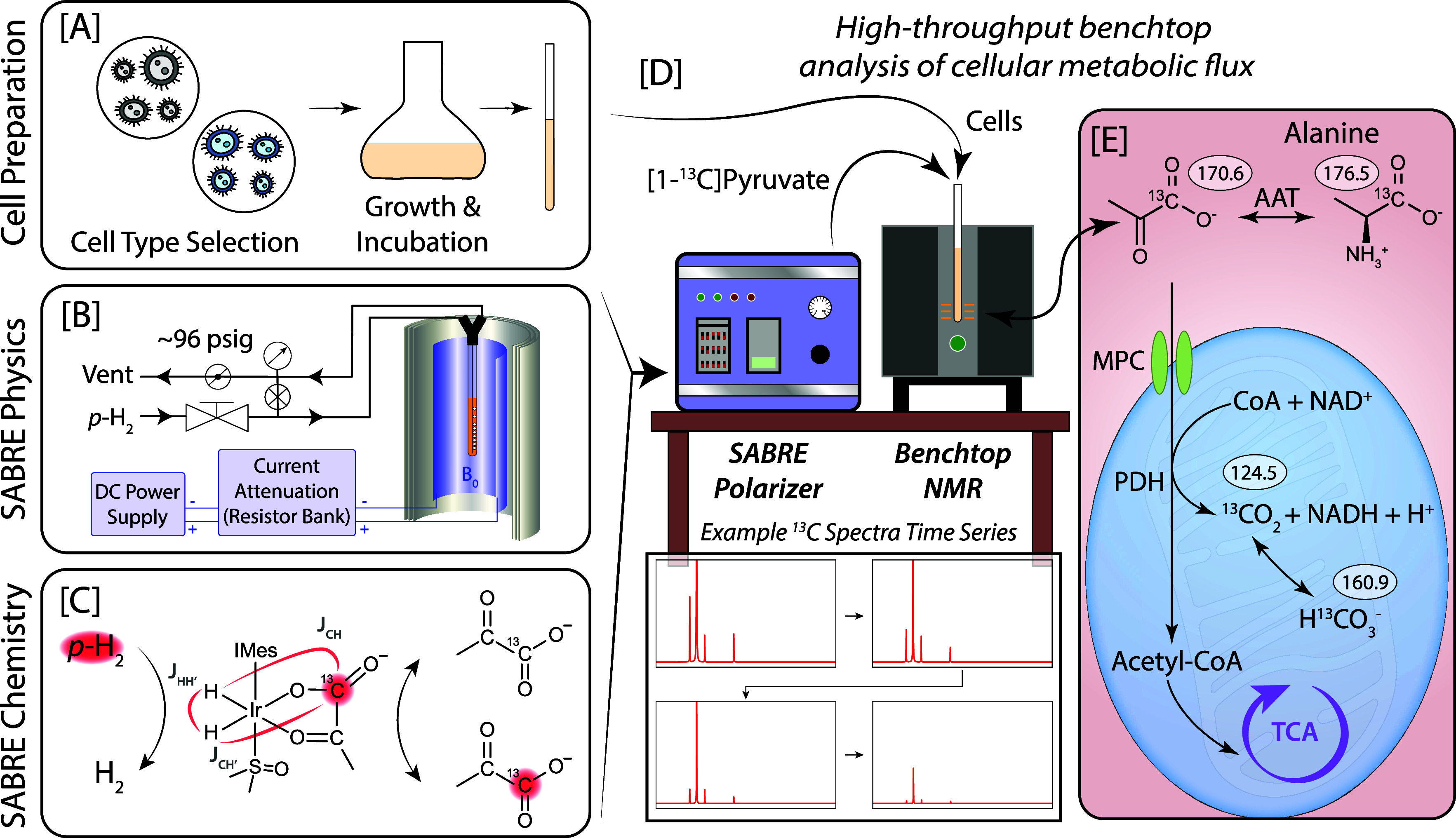
Graphical workflow for benchtop assessment of cellular metabolic flux with SABRE hyperpolarization. [A] Graphical depiction of the cell preparation process, where different cell subtypes could be used with the depicted benchtop workflow. [B] Bubbling setup used for SABRE hyperpolarization, where parahydrogen gas is regulated through an NMR tube in a shielded magnetic environment with an applied external field (typically 0.4 μT). [C] SABRE hyperpolarization chemistry, where parahydrogen and pyruvate exchange reversibly on an iridium metal catalyst (here, Ir-IMes), generating HP [1-^13^C]­pyruvate from transfer of the parahydrogen spin order to the pyruvate ^13^C nuclear spin. [D] Benchtop SABRE polarizer and NMR spectrometer, where injection of cells and HP pyruvate generates a time series of metabolic spectra. [E] Products of HP [1-^13^C]­pyruvate, showing the conversion of [1-^13^C]­pyruvate to [1-^13^C]­alanine via alanine aminotransferase (AAT) or shuttling into the mitochondria and TCA cycle for subsequent oxidative decarboxylation via aerobic metabolism (transport protein mitochondrial pyruvate carrier, MPC, and pyruvate dehydrogenase complex, PDH).

These features position SABRE as a highly accessible and scalable hyperpolarization strategy, especially for applications requiring repeated measurements across many samples, such as metabolic flux screening, pathway optimization in engineered bacteria, or high-throughput phenotyping in oncological cell lines.

Here, we present a SABRE-based platform that enables real-time tracking of metabolism in live-cell suspensions using HP [1-^13^C]­pyruvate (Figure 1). We demonstrate that this approach can resolve the conversion into downstream products, including [^13^C]­bicarbonate/^13^CO_2_ (oxidative decarboxylation), permitting kinetic visualization of metabolic flux. Using yeast as an exemplary system, we show tracking of the metabolic readout for over 3 min and acquisition with high temporal resolution using a 1.4 T benchtop NMR system without cryogenic equipment. The ability to observe cell metabolism with real-time resolution, nondestructive sampling, and native substrate labeling, positions SABRE HP pyruvate as a practical and powerful tool for biological research.

## Experimental Section

### Preparation and Hyperpolarization of [1-^13^C]­Pyruvate

A solution of [1-^13^C]­pyruvate for hyperpolarization via SABRE is prepared by dissolving 13.8 mg of an iridium-based SABRE precatalyst ([IrCl­(COD)­(IMes)], where COD = 1,5-cyclooctadiene and IMes = 1,3-bis­(2,4,6-trimethylphenyl)­imidazol-2-ylidene) and 15.5 mg [1-^13^C]­pyruvate (Cambridge Isotope Laboratories, Inc.) in 2.0 mL of an 80% (v/v) acetone (Fisher Scientific) in D_2_O (Cambridge Isotope Laboratories, Inc.) mixture, along with 24 mM dimethyl sulfoxide-d6 (DMSO-*d*_6_, Cambridge Isotope Laboratories, Inc.). Ir-IMes was synthesized as previously described.^45^ The mixture is placed in an ultrasonic bath until the catalyst and the substrate were completely dissolved, yielding a homogeneous solution. An aliquot of 1 mL of the solution is transferred into a regular 5 mm NMR tube fitted with a 1/4 in. PTFE tube (see SI for more details) as a reactor for hyperpolarization. To fully activate the catalyst, the NMR tube containing the sample is pressurized with 100 psi parahydrogen (*p*-H_2_) and placed into a cold-water bath (6.5 °C) situated in a SABRE polarizer operating at mictrotesla fields with a magnetic field of 0.40 mT (Figure 1B, see SI for more details).^37^ *p*-H_2_ (>98% enrichment, see SI) was bubbled through the solution at a flow rate of 100 sccm for 2 min intervals, acquiring the signal every 2 min until less than a 5% deviation in the sample signal was observed (see SI). After activation and stabilization of polarization, samples were subsequently polarized with 90 s of 100 sccm flow of parahydrogen through solution via a catheter, whereafter the NMR tube was either transferred for direct detection (*in situ*) in a 1.4 T benchtop NMR (SpinSolve Carbon, Magritek) or transferred to a separate fixture for preparation of the aqueous HP media.

### Preparation of Aqueous [1-^13^C]­Pyruvate Hyperpolarized Media

The biocompatible solution was prepared similarly to the protocol previously described by McBride et al.^44^ After hyperpolarization, the HP solution comprising [1-^13^C]­pyruvate, polarization catalyst, acetone, and D_2_O was moved to the surface of a custom one-sided Halbach array with a surface field of approximately 200 mT and the sample was depressurized. After depressurization, the *p*-H_2_ catheter was removed, a new catheter was inserted, and the HP solution was extracted into a syringe containing 4 mL of butyl acetate dyed with Sudan Blue II (Figure 2D, step 1). Upon mixing of the HP solution and butyl acetate in the syringe, a phase separation occurred, resulting in the formation of organic and aqueous phases. The aqueous phase in the bottom layer was then extracted via a clean catheter into a clean syringe (Figure 2D, step 2). This solution was then transferred to a Pyrex test tube heated to approximately 70 °C in a water bath to additionally purify the solution via flow of hydrogen gas through a catheter in the solution at a rate of 2.4 slm, further reducing the residual acetone content (Figure 2D, step 3). The aqueous HP pyruvate was then transferred into a syringe for analysis or metabolic sensing experiments (Figure 2D, step 4). The total time for the manual preparation of purified HP [1-^13^C]­pyruvate solutions was 50–55 s.

**Figure 2:**
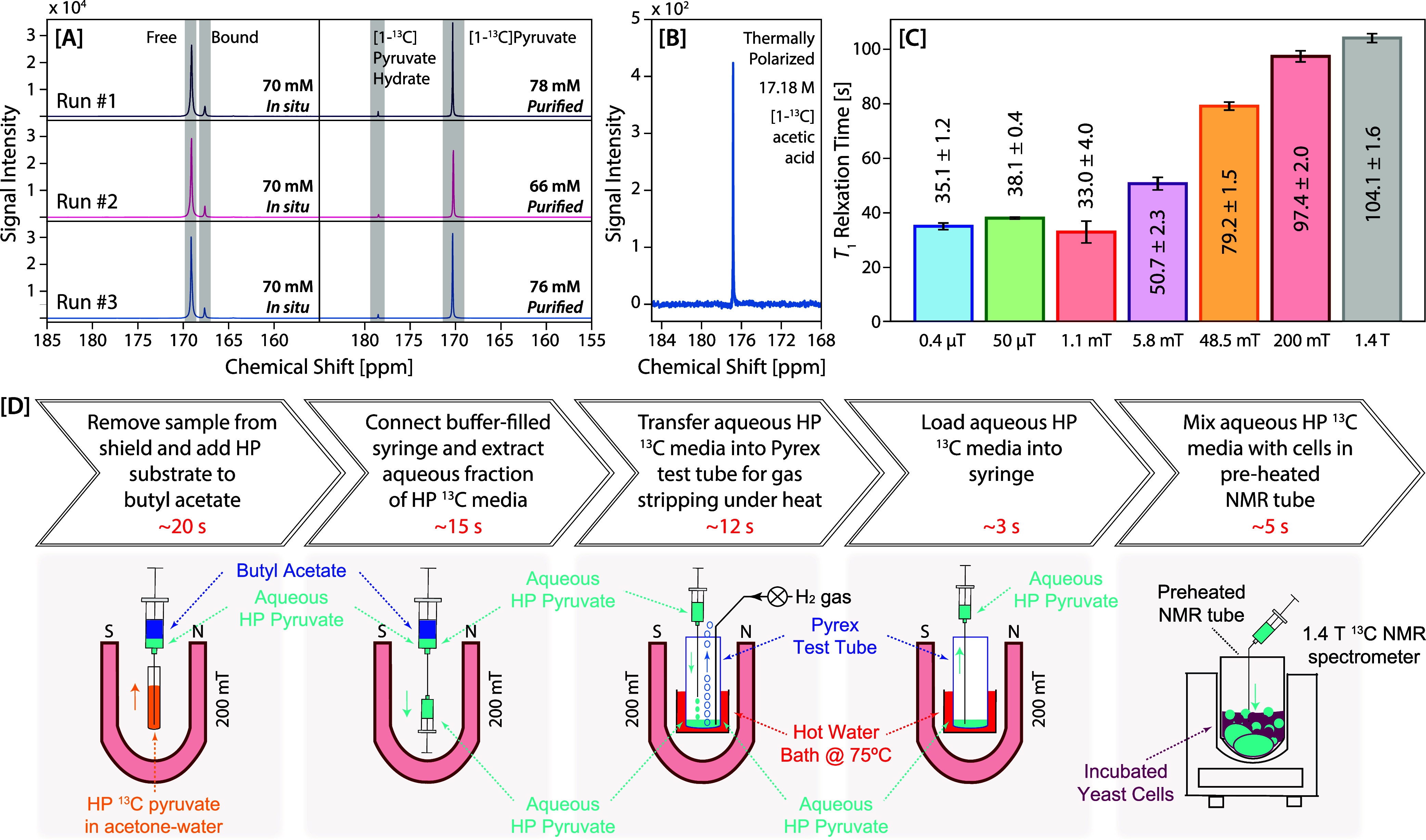
[A] Spectra from three separate SABRE HP [1-^13^C]­pyruvate samples, showing polarization both under hydrogen pressure, *in situ* in the 5 mm NMR tube reactor (left) and after processing for each respective sample (right). The difference in spectral line width between the *in situ* (left) and processed (right) spectra is potentially due to the dynamic exchange of the SABRE process and potential susceptibility artifacts from the bubbling catheter in the *in situ* sample, while the purified sample experiences no SABRE exchange. [B] Spectrum from a pure [1-^13^C]­acetic acid reference sample (17.18 M) acquired in a single shot. All spectra in [A] and [B] were acquired with a 7° flip angle on a 1.4 T SpinSolve Carbon benchtop NMR (Magritek). [C] Relaxation measurements *in situ* (in the hyperpolarization solution, under *p*-H_2_ pressure) at seven different magnetic fields. [D] Purification process used to transfer SABRE HP [1-^13^C]­pyruvate from an 80% acetone in D_2_O mixture into an aqueous solution, where the magnet loop represents the 0.2 T Halbach array.

### Metabolic Sensing of [1-^13^C]­Pyruvate in *Saccharomyces cerevisiae*

Yeast suspensions were prepared by homogenizing 7.0 g of *Saccharomyces cerevisiae* (Sc, Baker’s yeast) in 44.0 mL of 0.2 M phosphate buffer (Na_2_HPO_4_/NaH_2_PO_4_, pH ∼ 6.5) and incubating the mixture at 35 °C to activate cellular metabolism. The yeast suspension was spiked with 50 mM sucrose to serve as a carbon source to enhance cellular metabolism.^19^ Three minutes prior to an experiment where yeast metabolic flux was measured, 0.3 mL aliquots of the preincubated yeast suspension were transferred into 35 °C preheated high-throughput 5 mm NMR tubes (Wilmad Labglass) and placed into a 1.4 T benchtop NMR spectrometer (SpinSolve Carbon, Magritek). We did not measure the yeast cell count but included an estimate in the SI. After processing, approximately 0.3 mL of the HP aqueous [1-^13^C]­pyruvate solution prepared as described above was injected into the tube containing the yeast and homogenized by using a catheter to ensure even distribution. Metabolic flux was monitored by using a pseudo-2D ^13^C pulse sequence using a 7° excitation pulse with proton decoupling, an acquisition time of 1.6 s, and a repetition time (TR) of 3.5 s.

## Results and Discussion

### Reproducible Hyperpolarization and Processing of [1-^13^C]­Pyruvate

To emphasize the reproducibility of the biocompatible SABRE preparation, we show spectra in Figure 2A for measurements of the HP [1-^13^C]­pyruvate signal both *in situ* (in the 5 mm NMR tube reactor, under *p*-H_2_ pressure) and after translation into aqueous media. For the data presented, the samples are first polarized and measured *in situ*, and after the measurement of polarization, the samples are then purified using the method detailed above and shown in Figure 2D. The purified samples no longer show the bound pyruvate resonances but instead show both HP [1-^13^C]­pyruvate and HP [1-^13^C]­pyruvate hydrate resonances due to the pyruvate hydration equilibrium in aqueous solutions. For comparison and quantification (details in SI) of the polarization levels, a single shot spectrum of neat [1-^13^C]­acetic acid (17.18 M) is acquired and referenced (Figure 2B).

During purification of the HP samples, a one-sided 0.2 T Halbach magnet array is utilized. The magnet array helps to reduce the relaxation of the HP sample undergoing processing, as shown in Figure 2C. In these relaxation experiments, the HP sample is measured *in situ* in the 5 mm NMR tube reactor, prior to any processing (SABRE solution, 80% acetone in D_2_O). The data are acquired by first polarizing the sample, moving the sample to the relaxation field, and then transferring and/or acquiring the data in a 1.4 T benchtop NMR spectrometer (see SI). Here, the relaxation at both 0.4 μT (the polarization field) and Earth’s magnetic field (EMF) is shorter than external higher fields, where the Halbach array field relaxation and relaxation in the 1.4 T benchtop NMR are both approximately 100 s (Figure 2C). We hypothesize that this trend is due to the scalar couplings between the ^1^H and ^13^C spins in pyruvate dominating the relaxation mechanisms at lower fields, leading to relatively fast relaxation rates, while at higher fields the Zeeman splitting (chemical shift difference) between the pyruvate spins becomes greater than the scalar couplings, leading to increasingly longer relaxation times, where the relaxation plateaus or decreases at high magnetic fields due to chemical shift anisotropy effects. The trend shown in Figure 2C is supported by similar trends shown in previous works^29,46^ and exemplifies the need for maintaining the HP [1-^13^C]­pyruvate sample at a sufficiently high field to minimize the *T*_1_-induced depolarization of the HP state during the 50–55 s long purification process. Polarization losses observed are relatively aligned with primarily *T*_1_ mechanism loss.

Notably, the purification method used also leads to robust and reproducible results in the processed solutions, namely, in pyruvate concentration and residual acetone concentration. Table 1 shows the polarization levels, pyruvate concentrations, and acetone concentrations for the three runs shown in Figure 2A. Butyl acetate and iridium concentrations were not measured in this work due to measurements made in prior work showing negligible levels of butyl acetate (<1 mM) and less than 20 μM iridium.^44^ We envision that process automation of the purification procedure can reduce the total processing time by at least a factor of 2, significantly reducing the *T*_1_ depolarization losses. Moreover, future optimization of SABRE hyperpolarization can potentially increase ^13^C polarization by a factor of 2–4, using novel pulse methods or more efficient delivery of parahydrogen gas.^47–50^ Taken together, these future developments (ongoing work by the authors) are expected to yield biocompatible HP [1-^13^C]­pyruvate solutions with over 20% polarization.

**Table 1:** Composition of HP [1-^13^C]­Pyruvate Solution after Sample Processing (*n* = 3)

solution parameter	parameter values
initial polarization	8.6 ± 0.2%
final polarization	4.0 ± 0.3%
processed solution [1-^13^C]pyruvate concentration	74 ± 5 mM
processed solution residual acetone concentration	26 ± 3 mM

### Metabolic Sensing of Yeast Cells with SABRE HP [1-^13^C]­Pyruvate

To evaluate the metabolic sensitivity and biological applicability of SABRE-hyperpolarized [1-^13^C]­pyruvate, we conducted time-resolved NMR measurements of central energy metabolism in a suspension of *Saccharomyces cerevisiae*. Yeast was selected as a model system due to its metabolic robustness, rapid growth, and relevance across industrial biotechnology and biomedical research. A processed solution of SABRE HP [1-^13^C]­pyruvate was prepared and mixed with a suspension of actively respiring yeast cells in D_2_O (see Methods). The mixture was measured in a 1.4 T benchtop NMR spectrometer (SpinSolve Carbon, Magritek), starting approximately 3 s after mixing. This experiment was carried out in triplicate (n = 3) to show the reproducibility of this method, and all data was processed using singular value decomposition (SVD) and automated integration protocols (see SI for data processing details and all processed data). Figure 3A–D shows representative spectra at 35, 105, 175, and 336 s, respectively. These spectra reveal clear and temporally distinct signatures from multiple pyruvate-derived oxidative decarboxylation products: ^13^CO_2_ and [^13^C]­bicarbonate (via pyruvate dehydrogenase), and the hydrated form of pyruvate, [1-^13^C]­pyruvate hydrate.

**Figure 3:**
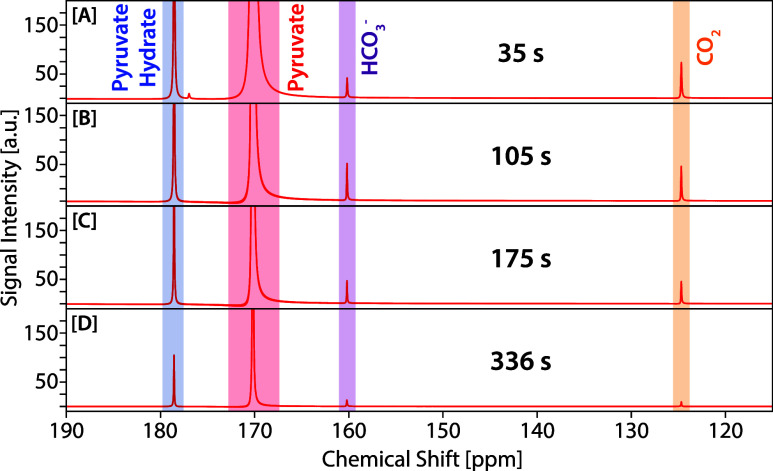
Example spectra of the metabolic and time resolution obtained in one experiment using HP [1-^13^C]­pyruvate to characterize metabolism in yeast cells. The experiment is conducted with spectra acquired every 3.5 s, and four example spectra are shown above. Here, the spectra shown are [A] 35 s, 10th transient, [B] 105 s, 30th transient, [C] 175 s, 50th transient, and [D] 336 s, 96th transient.

The resolution and quality of the spectra remain high throughout the experiment, demonstrating the compatibility of the SABRE method with live-cell suspensions and the capacity for extended monitoring windows. Importantly, these results exemplify several key strengths of the SABRE platform for benchtop metabolic analysis. First, using an entirely benchtop-based apparatus (polarizer and detector), we use the technique described to monitor metabolite signals over 5 min (particularly noting the detection of [^13^C]­bicarbonate and ^13^CO_2_ with signal-to-noise (SNR) ratios exceeding 10:1 at 336 s), underscoring how long-lived metabolic flux tracking is achievable with the use of low-field spectrometry combined with SABRE polarization. Second, the described technique generates biocompatible, polarized pyruvate in minutes, enabling the dissecting of distinct enzymatic pathways, including oxidative flux.

To extract quantitative insight into the metabolic dynamics of [1-^13^C]­pyruvate in live yeast cells, the time-resolved signals of each metabolite were fit to ordinary differential equation (ODE)-based models, utilizing a single decay constant (γ) to account for all sources of decay for the respective products. This approach leverages the strong initial polarization and high substrate concentration to treat pyruvate as a pseudosaturated reservoir that permits the treatment of the kinetics with first-order modeling of downstream products without back-coupling.^51^ The temporal profile of pyruvate hydrate was fit by using a single-compartment model governed by production and decay terms. In contrast, the oxidative decarboxylation products, CO_2_ and bicarbonate, were modeled using a two-compartment system to account for their interconversion through the carbonic acid buffer equilibrium, capturing both the direct enzymatic production of CO_2_ and subsequent conversion to bicarbonate.

The respective flows of pyruvate into these products are shown in Figure 4A. The average fits for all of the modeled data sets (*n* = 3) are given in Table 2, and exemplary fits for one data set are shown in Figure 4B–D (remaining data can be found in the SI). All models were fit using least-squares optimization, and further details of the differential equations and fitting constraints are provided in the SI.

**Figure 4:**
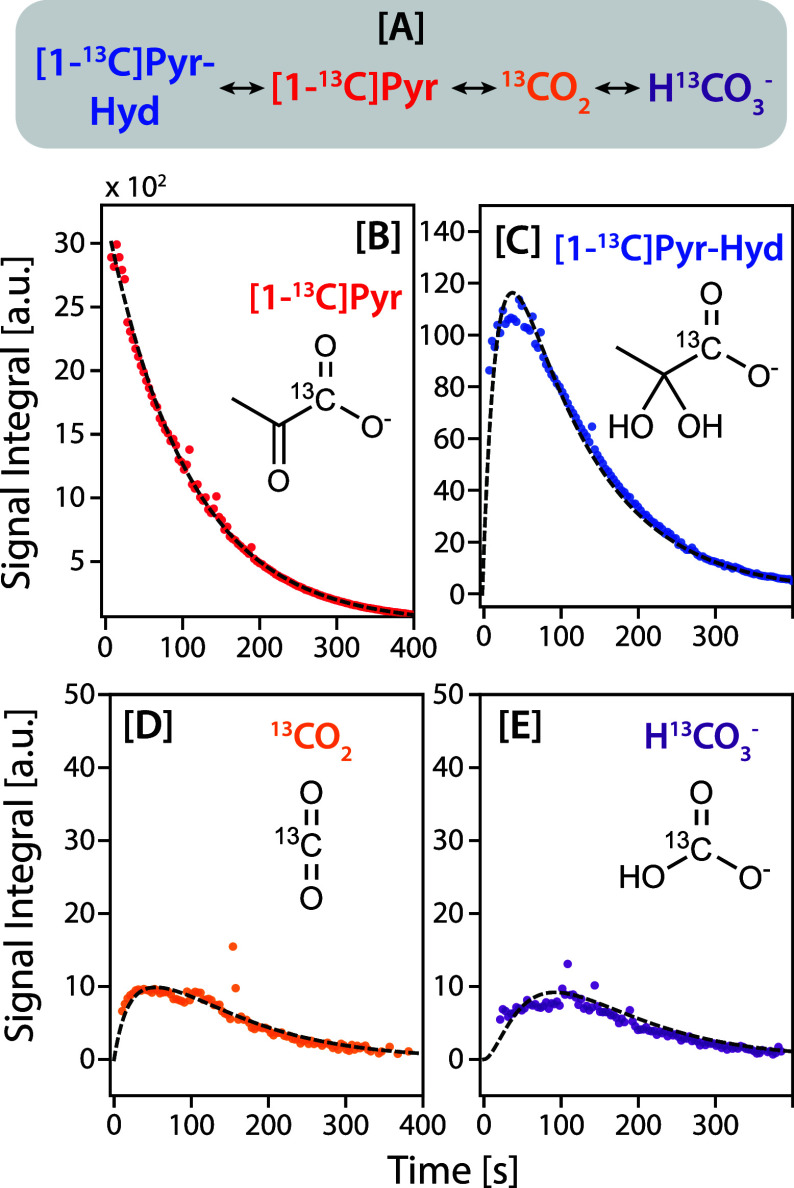
[A] Pathways of HP [1-^13^C]­pyruvate into the resulting products. Data and fittings of pyruvate and its products in one experiment: [B] pyruvate fit to a monoexponential decay and is used as the driving input function for the product kinetic models, [C] pyruvate hydrate fit to a one compartment ODE model, [D, E] CO_2_ and bicarbonate, respectively, fit to a two-compartment ODE model.

**Table 2:** Fitting Parameters for Kinetic Analysis of Pyruvate Metabolism in *Saccharomyces cerevisiae* (*n* = 3)

fitting parameter	parameter values
τ_PYR_	121 ± 14 s
*k*_PYR→CO_2__	0.00023 ± 0.00003 s^–1^
*k*_BIC→CO_2__	0.06 ± 0.01 s^–1^
*k*_CO_2_→BIC_	0.023 ± 0.003 s^–1^
*k*_PYR→PH_	0.0023 ± 0.0003 s^–1^
γ_PH_	0.052 ± 0.002
γ_BIC/CO_2__	0.015 ± 0.002

As evidenced by the monoexponential signal decay of HP [1-^13^C]­pyruvate (Figure 4B), pyruvate can be treated as a source term, where the fitted monoexponential decay (decay constant τ_PYR_) represents the substrate signal availability over time and is used as the driving input function in the kinetic models for downstream products. Pyruvate hydrate increases sharply due to the production initialization from pyruvate and then decays over the course of the experiment, consistent with first-order conversion kinetics. CO_2_ rises rapidly in the first minute of acquisition, reflecting the initial oxidative burst from pyruvate decarboxylation. Bicarbonate formation is delayed relative to that of CO_2_ but tracks upward in tandem once an equilibrium is established in the intracellular matrix.

Separately, the interconversion rates between CO_2_ and bicarbonate were found to be asymmetric, with faster forward hydration than reverse dehydration, consistent with expectations in mildly acidic environments.^52^ A potential advantage of this modeling framework is the ability to extract physiochemical insights from these relative signal dynamics. Specifically, we can utilize the derived fits for CO_2_ and bicarbonate to calculate the ratios of these products throughout the experiment. Here, we hypothesize that this ratio estimates the system’s apparent intracellular pH due to the agreement between the literature^52^ and the pH calculated from these ratios (we calculate an initial intracellular pH of 4.8 ± 0.1 at the start of acquisition, rising to 6.2 ± 0.1 by the end of the experiment). The pH evolution of the same experiment shown in Figure 4 is plotted in Figure 5 (all data are shown in the SI) alongside the HCO_3_^–^/CO_2_ signal ratio over time. Notably, these values differ from the bulk suspension pH measured before and after the experiment (5.9 and 6.4, respectively). Therefore, we hypothesize that the measured ratios and calculated pH reflect significant contributions from the intracellularly produced HP ^13^CO_2_ signal. This CO_2_ can be converted to bicarbonate either intra- or extracellularly, but the progressive shift toward extracellular pH over time likely reflects transport and equilibration of metabolites across the cell membrane. While more rigorous experiments would be needed to completely resolve these dynamics, the current results highlight the potential of this approach to noninvasively probe intracellular microenvironments.

**Figure 5:**
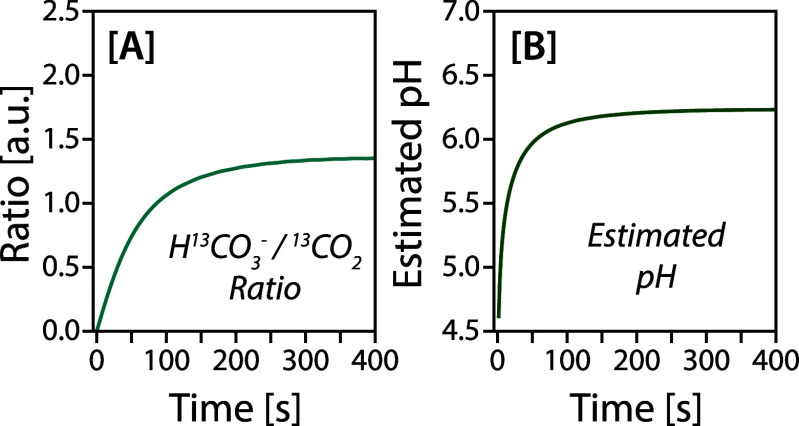
Comparison of bicarbonate and CO_2_ ratios derived from the fitted models [A] to estimate intracellular pH [B] using the Henderson–Hasselbach equation and the p*K*_a_ (6.1) of the carbonic acid buffer system.

## Outlook

Taken together, these results underscore the unique power of SABRE HP ^13^C spectroscopy to capture not only enzymatic flux but also dynamic biochemical buffering states from intact cells. The use of tailored ODE models allows distinct cell metabolism to be visualized and quantified with subminute resolution, offering a scalable and information-rich platform for cell-based metabolic phenotyping. This approach holds promise for screening of engineered microbial strains or perturbed mammalian cells, where both pathway activity and environmental context (e.g., intracellular pH and mitochondrial function) play critical roles in phenotype expression.

The scalability and simplicity of the SABRE platform make it well-suited for use in benchtop workflows to help drive future adoption across biomedical and biotechnological research. By enabling real-time, noninvasive tracking of cell metabolism, SABRE ^13^C-hyperpolarized metabolites offer a new method that could provide a tool for phenotyping and functional screening. In mammalian systems, including applications in oncology and immunometabolism, SABRE could be potentially applied for high-throughput versions of existing applications of HP pyruvate in monitoring of pyruvate-to-lactate conversion, oxidative metabolism, and redox state can inform therapy response and tumor profiling (ongoing work in our collaborating laboratories).^12,20,24,32,53^ In microbial systems, SABRE HP-based metabolic analysis could potentially be applied to accelerate pathway optimization and strain engineering, offering a new tool to identify (for example) flux bottlenecks in engineering *Escherichia coli* for antibiotic production^54,55^ or *Saccharomyces cerevisiae* for ethanol and butanol production.^56^

## Conclusion

This work demonstrates the development of SABRE-based HP [1-^13^C]­pyruvate as a benchtop platform for cellular metabolic sensing. Our results with *Saccharomyces cerevisiae* establish key capabilities of this platform, enabling visualization of cell metabolism with subminute temporal resolution over extended timeframes (>3 min). The reproducible hyperpolarization levels and biocompatible purification protocol demonstrate the method’s reliability for routine use in living cells in an entirely benchtop workflow. Beyond simple flux measurements, the analysis of CO_2_ and bicarbonate product ratios enables real-time estimation of pH dynamics. This capability to probe cellular microenvironments directly and noninvasively represents a significant advancement in cellular metabolic sensing technology. This work represents the first step in establishing a SABRE-based platform as a benchtop, generalized lab platform to perform metabolic flux measurements in cell-based systems. The combination of rapid polarization cycles, room-temperature operation, and benchtop workflow leverages the accessibility and scalability of SABRE hyperpolarization with benchtop NMR detection to make this approach uniquely suited for future widespread adoption in high-throughput applications across diverse fields, from drug screening to pathway optimization in engineered microorganisms.

## Supplementary Material



## Data Availability

The data underlying this study are available in the published article and online Supporting Information. The raw NMR spectroscopic data is available in Mendeley repository under TomHon, Patrick (2026), “Metabolite Sensing in Yeast Cells with SABRE-Hyperpolarized [1–^13^C]­Pyruvate”, Mendeley Data, V1, doi: 10.17632/mx35mhb5td.1.

## Notes

Additional experimental details, materials, sample characterization, and data processing methods (PDF)

## References

[1] Qiu S., Cai Y., Yao H., Lin C., Xie Y., Tang S., Zhang A. (2023). Small Molecule Metabolites: Discovery of Biomarkers and Therapeutic
Targets. Signal Transduction Targeted Ther..

[2] Gonzalez-Covarrubias V., Martínez-Martínez E., del Bosque-Plata L. (2022). The Potential
of Metabolomics in Biomedical Applications. Metabolites.

[3] Zampieri M., Sekar K., Zamboni N., Sauer U. (2017). Frontiers of High-Throughput
Metabolomics. Curr. Opin. Chem. Biol..

[4] Collins S. L., Koo I., Peters J. M., Smith P. B., Patterson A. D. (2021). Current
Challenges and Recent Developments in Mass Spectrometry–Based
Metabolomics. Annu. Rev. Anal. Chem..

[5] Zaikin V. G., Borisov R. S. (2021). Mass Spectrometry as a Crucial Analytical Basis for
Omics Sciences. J. Anal. Chem..

[6] Gooz M., Maldonado E. N. (2023). Fluorescence
Microscopy Imaging of Mitochondrial Metabolism
in Cancer Cells. Front. Oncol..

[7] Ji W., Tang X., Du W., Lu Y., Wang N., Wu Q., Wei W., Liu J., Yu H., Ma B., Li L., Huang W. (2022). Optical/Electrochemical Methods for Detecting Mitochondrial
Energy Metabolism. Chem. Soc. Rev..

[8] Sewell F., Alexander-White C., Brescia S., Currie R. A., Roberts R., Roper C., Vickers C., Westmoreland C., Kimber I. (2024). New Approach Methodologies
(NAMs): Identifying and
Overcoming Hurdles to Accelerated Adoption. Toxicol. Res..

[9] Levine S. L., Riter L. S., Lagadic L., Bejarano A. C., Burden N., Burgoon L. D., Becker R. A., Ryman J. P., Armbrust K. L. (2025). Challenges
and Opportunities in the Development and Adoption of New Approach
Methods (NAMs). J. Agric. Food Chem..

[10] Ardenkjær-Larsen J. H., Fridlund B., Gram A., Hansson G., Hansson L., Lerche M. H., Servin R., Thaning M., Golman K. (2003). Increase in
Signal-to-Noise Ratio of > 10,000 Times in Liquid-State NMR. Proc. Natl. Acad. Sci. U.S.A..

[11] Hövener J., Pravdivtsev A. N., Kidd B., Bowers C. R., Glöggler S., Kovtunov K. V., Plaumann M., Katz-Brull R., Buckenmaier K., Jerschow A., Reineri F., Theis T., Shchepin R. V., Wagner S., Bhattacharya P., Zacharias N. M., Chekmenev E. Y. (2018). Parahydrogen-Based Hyperpolarization
for Biomedicine. Angew. Chem., Int. Ed..

[12] Vaeggemose M., F Schulte R., Laustsen C. (2021). Comprehensive Literature Review of
Hyperpolarized Carbon-13 MRI: The Road to Clinical Application. Metabolites.

[13] Merritt M. E., Harrison C., Storey C., Jeffrey F. M., Sherry A. D., Malloy C. R. (2007). Hyperpolarized 13C
Allows a Direct Measure of Flux
through a Single Enzyme-Catalyzed Step by NMR. Proc. Natl. Acad. Sci. U.S.A..

[14] Nelson S. J., Kurhanewicz J., Vigneron D. B., Larson P. E. Z., Harzstark A. L., Ferrone M., van Criekinge M., Chang J. W., Bok R., Park I., Reed G., Carvajal L., Small E. J., Munster P., Weinberg V. K., Ardenkjaer-Larsen J. H., Chen A. P., Hurd R. E., Odegardstuen L.-I., Robb F. J., Tropp J., Murray J. A. (2013). Metabolic Imaging
of Patients with Prostate Cancer Using Hyperpolarized [1-^13^C]­Pyruvate. Sci. Transl. Med..

[15] Hu S., Balakrishnan A., Bok R. A., Anderton B., Larson P. E. Z., Nelson S. J., Kurhanewicz J., Vigneron D. B., Goga A. (2011). 13C-Pyruvate
Imaging Reveals Alterations in Glycolysis That Precede c-Myc-Induced
Tumor Formation and Regression. Cell Metab..

[16] Takakusagi Y., Takakusagi K., Inoue K., Saito K., Homma Y., Ichikawa K. (2025). Hyperpolarized [1–13C]­Pyruvate NMR Spectroscopy
Reveals Transition of Tumor Energy Metabolism in Microscale Multicellular
Spheroids. Sci. Rep..

[17] Harris T., Eliyahu G., Frydman L., Degani H. (2009). Kinetics of Hyperpolarized
13C1-Pyruvate Transport and Metabolism in Living Human Breast Cancer
Cells. Proc. Natl. Acad. Sci. U.S.A..

[18] Sriram R., Nguyen J., Santos J. D., Nguyen L., Sun J., Vigneron S., Van Criekinge M., Kurhanewicz J., MacKenzie J. D. (2018). Molecular Detection of Inflammation in Cell Models
Using Hyperpolarized 13C-Pyruvate. Theranostics.

[19] Peters J.
P., Assaf C., Mohamad F. H., Beitz E., Tiwari S., Aden K., Hövener J.-B., Pravdivtsev A. N. (2024). Yeast Solutions
and Hyperpolarization Enable Real-Time Observation of Metabolized
Substrates Even at Natural Abundance. Anal.
Chem..

[20] Bliemsrieder E., Kaissis G., Grashei M., Topping G., Altomonte J., Hundshammer C., Lohöfer F., Heid I., Keim D., Gebrekidan S., Trajkovic-Arsic M., Winkelkotte A. M., Steiger K., Nawroth R., Siveke J., Schwaiger M., Makowski M., Schilling F., Braren R. (2021). Hyperpolarized 13C
Pyruvate Magnetic Resonance Spectroscopy for in Vivo Metabolic Phenotyping
of Rat HCC. Sci. Rep..

[21] Hyppönen V., Stenroos P., Nivajärvi R., Ardenkjær-Larsen J. H., Gröhn O., Paasonen J., Kettunen M. I. (2022). Metabolism of Hyperpolarised
[1–13C]­Pyruvate in Awake and Anaesthetised Rat Brains. NMR Biomed..

[22] Josan S., Park J. M., Hurd R., Yen Y.-F., Pfefferbaum A., Spielman D., Mayer D. (2013). In Vivo Investigation
of Cardiac
Metabolism in the Rat Using MRS of Hyperpolarized [1–13C] and
[2–13C]­Pyruvate. NMR Biomed..

[23] Choi Y.-S., Kang S., Ko S.-Y., Lee S., Kim J. Y., Lee H., Song J. E., Kim D.-H., Kim E., Kim C. H., Saksida L., Song H.-T., Lee J. E. (2018). Hyperpolarized
[1–13C]
Pyruvate MR Spectroscopy Detect Altered Glycolysis in the Brain of
a Cognitively Impaired Mouse Model Fed High-Fat Diet. Mol. Brain.

[24] Dou Q., Grant A. K., Coutinto
de Souza P., Moussa M., Nasser I., Ahmed M., Tsai L. L. (2024). Characterizing Metabolic Heterogeneity
of Hepatocellular Carcinoma with Hyperpolarized 13C Pyruvate MRI and
Mass Spectrometry. Radiol. Imaging Cancer.

[25] Kjaergaard U., Lund A., Redda M., Kristensen M. H., Aastrup M., Bøgh N., Sivesgaard K., Ohliger M. A., Vigneron D. B., Bertelsen L. B., Alstrup A. K. O., Hansen E. S. S., Mortensen F. V., Laustsen C. (2025). Regional Quantification of Metabolic Liver Function
Using Hyperpolarized [1–13C] Pyruvate MRI. Sci. Rep..

[26] Sharma G., Enriquez J. S., Armijo R., Wang M., Bhattacharya P., Pudakalakatti S. (2023). Enhancing
Cancer Diagnosis with Real-Time Feedback:
Tumor Metabolism through Hyperpolarized 1–13C Pyruvate MRSI. Metabolites.

[27] Larson P. E. Z., Bernard J. M. L., Bankson J. A., Bøgh N., Bok R. A., Chen A. P., Cunningham C. H., Gordon J. W., Hövener J.-B., Laustsen C., Mayer D., McLean M. A., Schilling F., Slater J. B., Vanderheyden J.-L., von Morze C., Vigneron D. B., Xu D. (2024). Current Methods for
Hyperpolarized [1–13C]­Pyruvate MRI Human Studies. Magn. Reson. Med..

[28] Salnikov O. G., Chukanov N. V., Pravdivtsev A. N., Burueva D. B., Sviyazov S. V., Them K., Hövener J.-B., Koptyug I. V. (2025). Heteronuclear Parahydrogen-Induced
Hyperpolarization via Side Arm Hydrogenation. ChemPhysChem.

[29] Nagel L., Gierse M., Gottwald W., Ahmadova Z., Grashei M., Wolff P., Josten F., Karaali S., Müller C. A., Lucas S., Scheuer J., Müller C., Blanchard J., Topping G. J., Wendlinger A., Setzer N., Sühnel S., Handwerker J., Vassiliou C., van Heijster F. H. A., Knecht S., Keim M., Schilling F., Schwartz I. (2023). Parahydrogen-Polarized [1–13C]­Pyruvate
for Reliable and Fast Preclinical Metabolic Magnetic Resonance Imaging. Adv. Sci..

[30] Cavallari E., Carrera C., Sorge M., Bonne G., Muchir A., Aime S., Reineri F. (2018). The 13C Hyperpolarized Pyruvate Generated
by ParaHydrogen Detects the Response of the Heart to Altered Metabolism
in Real Time. Sci. Rep..

[31] Adams R. W., Aguilar J. A., Atkinson K. D., Cowley M. J., Elliott P. I. P., Duckett S. B., Green G. G. R., Khazal I. G., López-Serrano J., Williamson D. C. (2009). Reversible Interactions with Para-Hydrogen
Enhance
NMR Sensitivity by Polarization Transfer. Science.

[32] Schmidt A. B., Chekmenev E. Y., de Maissin H., Groß P. R., Petersen S., Nagel L., Schilling F., Schwartz I., Reinheckel T., Hövener J.-B., Knecht S. (2024). Signal Amplification by Reversible Exchange and Its
Translation to Hyperpolarized Magnetic Resonance Imaging in Biomedicine. Anal. Sens..

[33] Barskiy D. A., Knecht S., Yurkovskaya A. V., Ivanov K. L. (2019). SABRE: Chemical
Kinetics and Spin Dynamics of the Formation of Hyperpolarization. Prog. Nucl. Magn. Reson. Spectrosc..

[34] Iali W., Roy S. S., Tickner B. J., Ahwal F., Kennerley A. J., Duckett S. B. (2019). Hyperpolarising
Pyruvate through Signal Amplification
by Reversible Exchange (SABRE). Angew. Chem.,
Int. Ed..

[35] Adelabu I., TomHon P., Kabir M. S. H., Nantogma S., Abdulmojeed M., Mandzhieva I., Ettedgui J., Swenson R. E., Krishna M. C., Theis T., Goodson B. M., Chekmenev E. Y. (2022). Order-Unity
13C Nuclear Polarization of [1–13C]­Pyruvate in Seconds and
the Interplay of Water and SABRE Enhancement. ChemPhysChem.

[36] TomHon P., Abdulmojeed M., Adelabu I., Nantogma S., Kabir M. S. H., Lehmkuhl S., Chekmenev E. Y., Theis T. (2022). Temperature Cycling
Enables Efficient 13C SABRE-SHEATH Hyperpolarization and Imaging of
[1–13C]-Pyruvate. J. Am. Chem. Soc..

[37] Nantogma S., Chowdhury M. R. H., Kabir M. S. H., Adelabu I., Joshi S. M., Samoilenko A., de Maissin H., Schmidt A. B., Nikolaou P., Chekmenev Y. A., Salnikov O. G., Chukanov N. V., Koptyug I. V., Goodson B. M., Chekmenev E. Y. (2024). MATRESHCA: Microtesla Apparatus for
Transfer of Resonance Enhancement of Spin Hyperpolarization via Chemical
Exchange and Addition. Anal. Chem..

[38] Theis T., Truong M. L., Coffey A. M., Shchepin R. V., Waddell K. W., Shi F., Goodson B. M., Warren W. S., Chekmenev E. Y. (2015). Microtesla
SABRE Enables 10% Nitrogen-15 Nuclear Spin Polarization. J. Am. Chem. Soc..

[39] Truong M. L., Theis T., Coffey A. M., Shchepin R. V., Waddell K. W., Shi F., Goodson B. M., Warren W. S., Chekmenev E. Y. (2015). 15N Hyperpolarization
by Reversible Exchange Using SABRE-SHEATH. J.
Phys. Chem. C.

[40] Tickner B. J., Semenova O., Iali W., Rayner P. J., Whitwood A. C., Duckett S. B. (2020). Optimisation of
Pyruvate Hyperpolarisation Using SABRE
by Tuning the Active Magnetisation Transfer Catalyst. Catal. Sci. Technol..

[41] Tickner B. J., Ahwal F., Whitwood A. C., Duckett S. B. (2021). Reversible Hyperpolarization
of Ketoisocaproate Using Sulfoxide-containing Polarization Transfer
Catalysts. ChemPhysChem.

[42] de
Maissin H., Groß P. R., Mohiuddin O., Weigt M., Nagel L., Herzog M., Wang Z., Willing R., Reichardt W., Pichotka M., Heß L., Reinheckel T., Jessen H. J., Zeiser R., Bock M., von Elverfeldt D., Zaitsev M., Korchak S., Glöggler S., Hövener J.-B., Chekmenev E. Y., Schilling F., Knecht S., Schmidt A. B. (2023). In Vivo Metabolic Imaging of [1–13C]­Pyruvate-D3
Hyperpolarized By Reversible Exchange With Parahydrogen. Angew. Chem., Int. Ed..

[43] Schmidt A. B., de Maissin H., Adelabu I., Nantogma S., Ettedgui J., TomHon P., Goodson B. M., Theis T., Chekmenev E. Y. (2022). Catalyst-Free
Aqueous Hyperpolarized [1–13C]­Pyruvate Obtained by Re-Dissolution
Signal Amplification by Reversible Exchange. ACS Sens..

[44] McBride S. J., Pike M., Curran E., Zavriyev A., Adebesin B., Tucker L., Harzan J. M., Senanayake I. M., Shen S., Abdulmojeed M., Theiss F., Boele T., Gade T. P., Duckett S., Goodson B. M., Rosen M. S., Chekmenev E. Y., Yuan H., Dedesma C., Kadlecek S., Theis T., TomHon P. (2025). Scalable Hyperpolarized MRI Enabled
by Ace-SABRE of [1–13C]­Pyruvate. Angew.
Chem., Int. Ed..

[45] Cowley M. J., Adams R. W., Atkinson K. D., Cockett M. C. R., Duckett S. B., Green G. G. R., Lohman J. A. B., Kerssebaum R., Kilgour D., Mewis R. E. (2011). Iridium N-Heterocyclic Carbene Complexes
as Efficient Catalysts for Magnetization Transfer from Para-Hydrogen. J. Am. Chem. Soc..

[46] Browning A., Macculloch K., TomHon P., Mandzhieva I., Chekmenev E. Y., Goodson B. M., Lehmkuhl S., Theis T. (2023). Spin Dynamics
of [1,2–13C2]­Pyruvate Hyperpolarization by Parahydrogen in
Reversible Exchange at Micro Tesla Fields. Phys.
Chem. Chem. Phys..

[47] Duchowny A., Denninger J., Lohmann L., Theis T., Lehmkuhl S., Adams A. (2023). SABRE Hyperpolarization
with up to 200 bar Parahydrogen in Standard
and Quickly Removable Solvents. Int. J. Mol.
Sci..

[48] Eriksson S. L., Mammen M. W., Eriksson C. W., Lindale J. R., Warren W. S. (2022). Multiaxial
Fields Improve SABRE Efficiency by Preserving Hydride Order. J. Magn. Reson..

[49] Schmidt A. B., Eills J., Dagys L., Gierse M., Keim M., Lucas S., Bock M., Schwartz I., Zaitsev M., Chekmenev E. Y., Knecht S. (2023). Over 20% Carbon-13
Polarization of
Perdeuterated Pyruvate Using Reversible Exchange with Parahydrogen
and Spin-Lock Induced Crossing at 50 μT. J. Phys. Chem. Lett..

[50] Kozinenko V. P., Kiryutin A. S., Yurkovskaya A. V. (2025). SLIC-SABRE
at Microtesla Fields Enables
High Levels of Nuclear Spin Polarization Without Magnetic Shielding. Chem. – Methods.

[51] Smallbone K., Messiha H. L., Carroll K. M., Winder C. L., Malys N., Dunn W. B., Murabito E., Swainston N., Dada J. O., Khan F., Pir P., Simeonidis E., Spasić I., Wishart J., Weichart D., Hayes N. W., Jameson D., Broomhead D. S., Oliver S. G., Gaskell S. J., McCarthy J. E. G., Paton N. W., Westerhoff H. V., Kell D. B., Mendes P. (2013). A Model of Yeast Glycolysis Based
on a Consistent Kinetic Characterisation of All Its Enzymes. FEBS Lett..

[52] Elsutohy M. M., Chauhan V. M., Markus R., Kyyaly M. A., Tendler S. J. B., Aylott J. W. (2017). Real-Time Measurement
of the Intracellular pH of Yeast
Cells during Glucose Metabolism Using Ratiometric Fluorescent Nanosensors. Nanoscale.

[53] Sunassee E. D., Jardim-Perassi B. V., Madonna M. C., Ordway B., Ramanujam N. (2023). Metabolic
Imaging as a Tool to Characterize Chemoresistance and Guide Therapy
in Triple-Negative Breast Cancer (TNBC). Mol.
Cancer Res..

[54] Homer J. A., Johnson R. M., Koelln R. A., Moorhouse A. D., Moses J. E. (2025). Strategic Re-Engineering of Antibiotics. Nat. Rev. Bioeng..

[55] Jeyachandran S., Vibhute P., Kumar D., Ragavendran C. (2024). Random Mutagenesis
as a Tool for Industrial Strain Improvement for Enhanced Production
of Antibiotics: A Review. Mol. Biol. Rep..

[56] Shi S., Chen Y., Nielsen J. (2025). Metabolic
Engineering of Yeast. Annu. Rev. Biophys..

