# Supplementary material for: Robust Rapid Cellular Metabolite Sensing Using Benchtop NMR and SABRE-Hyperpolarized [1-13C]Pyruvate
Source: Anal Chem. 2026 Mar 10;98(11):8051–9. doi: 10.1021/acs.analchem.5c05076 (PMC13019433; doi:10.1021/acs.analchem.5c05076)
Supplement: Supplementary file 1 [file ac5c05076_si_001.pdf]

SUPPORTING INFORMATION

for

Robust Rapid Benchtop NMR Cellular Metabolite Sensing Using SABRE-Hyperpolarized [1-^{13}C]Pyruvate

Joseph Gyesi,[a+] Patrick TomHon,[a+*] Abubakar Abdurraheem,[a] Anna Samoilenko,[a] Sydney Scofield,[b] Clementinah Oladun,[a] Stephen McBride,[c] Erica Curran,[c] Megan Pike,[c] Kamal Kadari,[a] Sydney D. Welch,[a] Sam Lipka,[a] Steven Balboa,[a] Charlie Fehl,[a] Marianna Sadagurski,[b] Jan-Bernd Hövener,[d] Thomas Theis,[c] Boyd M. Goodson,[e] and Eduard Y. Chekmenev[a*]

[a] Department of Chemistry, Integrative Biosciences (Ibio), Karmanos Cancer Institute (KCI), Wayne State University, Detroit, Michigan 48202, United States
[b] Biological Sciences, Center for Molecular Medicine and Genetics, Wayne State University, Detroit, Michigan 48202, United States
[c] Department of Chemistry, North Carolina State University, Raleigh, North Carolina 27606, United States
[d] Section Biomedical Imaging, Molecular Imaging North Competence Center (MOIN CC), Department of Radiology and Neuroradiology, University Medical Center Kiel, Kiel University, Am Botanischen Garten 18, Kiel 24118, Germany
[e] School of Chemical & Biomolecular Sciences and Materials Technology Center, Southern Illinois University, Carbondale, Illinois 62901, United States

[+] *J.G. and P.T. contributed equally to this work.*

Table of Contents

1. Introduction....3
2. Sample Preparation and NMR Data Acquisition....3
2.1. [1-^{13}C]pyruvate sample preparation....3
2.2. Parahydrogen Preparation....3
2.3. Hyperpolarization experiments....3
2.4. Polarization Quantification....6
3. Experiment Details....7
3.1. Effective T_1 Relaxation Experiments....7
3.2. Hyperpolarized Solution Processing....10
3.2.1 Characterization of Processed Hyperpolarized [1-^{13}C]Pyruvate Solutions....10
3.3. Cellular Metabolic Profiling Experiments....12
3.3.1 Cell Preparation and Activation....12
3.3.2 Metabolic Profiling Measurement Protocol....13
4. Data Analysis and Processing of Cellular Metabolic Experiments....13
4.1. De-noising of Raw Signals with Singular Value Decomposition (SVD)....13
4.2. Analysis of Cellular Metabolism with Ordinary Differential Equation (ODE) Models....25
4.3. Intracellular pH Estimation using Metabolite Fitting....27
5. Supporting Information References....28

1. Introduction

The first section of the supporting information (SI) describes the preparation of samples, acquisition of NMR data, and calculation of polarization values. The second section describes the details of specific experiments, including the T_1 relaxation experiments, processing of hyperpolarized pyruvate solutions, and the calculation of acetone and pyruvate concentrations in the processed solutions. The third section describes the preparation of cells for metabolic profiling experiments and the data processing used for de-noising and fitting of this data.

2. Sample Preparation and NMR Data Acquisition

2.1. [1-^{13}C]pyruvate sample preparation

Experimental samples were prepared by first pre-weighing 13.8 mg of an iridium-based SABRE pre-catalyst ([IrCl(COD)(IMes)], where COD = 1,5-cyclooctadiene and IMes = 1,3-bis(2,4,6-trimethylphenyl)imidazol-2-ylidene)[1,2] and 15.5 mg of [1-^{13}C]pyruvate, sodium salt (Cambridge Isotope Laboratories, CLM-1082) in a 2.0 mL vial and capped with argon gas (99.999%). Separately, a solution of 200 mM DMSO-d6 (Cambridge Isotope Laboratories, DLM-10-10X0.6) in D_2O (Cambridge Isotope Laboratories, DLM-4) was prepared under argon gas. Subsequently, 240 µL of 200 mM DMSO in D_2O solution along with 160 µL D_2O and 1600 µL acetone (Fisher Scientific HPLC Grade, A949) were added to the pre-weighed SABRE pre-catalyst and [1-^{13}C]pyruvate. The resulting solution as sonicated until the material was completely dissolved, yielding a 70 mM concentration of [1-^{13}C]pyruvate and 10.8 mM concentration of SABRE pre-catalyst. All solvents were separately degassed with argon prior to solution and sample formulation.

2.2. Parahydrogen Preparation

Parahydrogen (p-H_2) was produced using enrichment at 25K using a commercially available parahydrogen generator (Advanced Research Systems, Inc.) and stored in a aluminum tank (~10 L), yielding a parahydrogen fraction of 95-98%.[3]

2.3. Hyperpolarization experiments

Formulated samples were pressurized under 120 psi of p-H_2 in a 5 mm medium wall high-throughput NMR tube (Wilmad Labglass). Samples were then placed in a 6.5°C cooling bath (based on previous work[4,5]). While in the cooling bath, p-H_2 was bubbled through the solution in 2 minute intervals (100 sccm) to activate the sample via a catheter in the NMR tube. The hyperpolarized signal was acquired every 2 minutes to check the signal level during this activation process, and the activation process was completed after less than a 5% deviation was observed in the sample signal level. This activation process was typically completed in 4-5 cycles (8-10 minutes) and **Table S1** shows an example of activation buildup.

Table S1. HP signal integral and P_{13C} during sample activation for an exemplary sample.

Cycle #	[1-^{13}C]Pyruvate Signal Integral	P_{13C} (15 MHz)	P_{13C} Change
1	1.00	6.22	N/A
2	1.40	8.69	28.4%
3	1.77	10.99	20.9%
4	1.76	10.98	-0.1%

After activation, all hyperpolarization experiments were conducted with 90 seconds of 100 sccm p-H_2 bubbling through the catheter at 6.5°C and 0.4 μT. The experimental setup used for these experiments and the activation above has been previously described (**Figure S1**).[6] All NMR experiments were detected with a 7.0-degree pulse in a 1.4 T benchtop NMR spectrometer (SpinSolve Carbon, Magritek). A 7.0-degree flip angle was used in all experiments due to the high signal in hyperpolarization experiments to minimize non-linear radiation dampening effects.

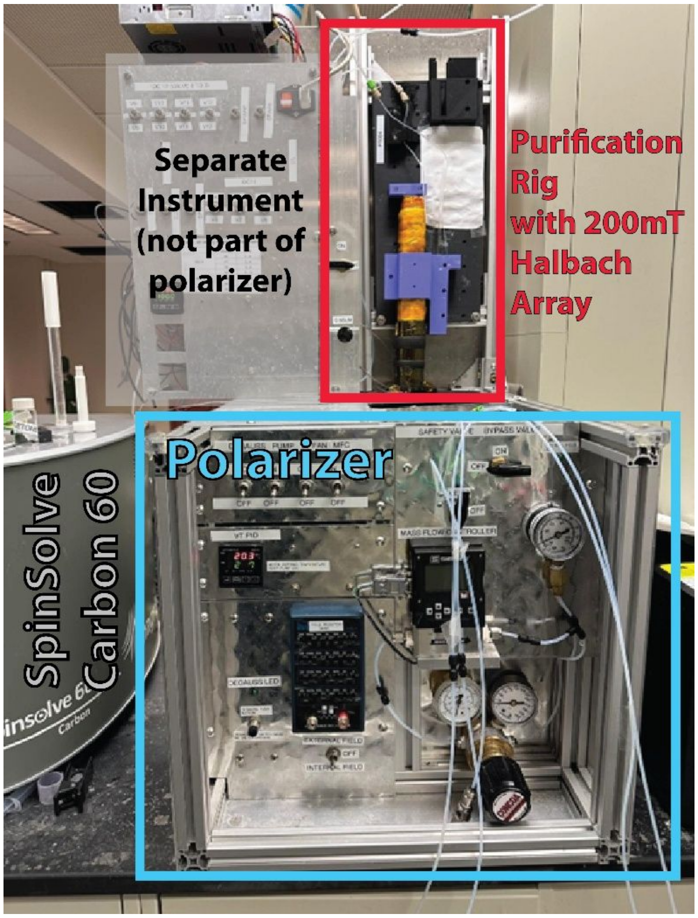

Figure S1. Picture of the experimental setup used to hyperpolarize samples. For more information about the polarizer, see prior publication.[6]

To characterize the magnetic field profile of [1-^{13}C]pyruvate in the polarizer setup above to ensure accuracy of the field used for polarization transfer, we performed a sweep of the magnetic field. This was performed by varying the resistance on the resistor bank shown in **Figure S1**, varying the magnetic field from 0.114 to 0.685 μT. The results of this sweep are shown below in **Table S2** and **Figure S2**. Here, an optimum is found at ~0.35 μT, which is rounded to 0.4 μT for all the applied work.

Table S2. Normalized HP signal integrals at each relaxation field.

Resistance [kΩ]	Field [μT]	Integral
27	0.685	0.1902
30	0.614	0.2475
32	0.575	0.3619
35	0.525	0.454
38	0.483	0.5293
40	0.46	0.5984
41	0.449	0.6011
42	0.438	0.6235
43	0.427	0.6388
44	0.417	0.6622
45	0.407	0.694
46	0.399	0.6935
47	0.39	0.6998
50	0.366	0.7033
55	0.331	0.7093
60	0.303	0.6754
65	0.279	0.6538
70	0.257	0.6041
75	0.24	0.5236
80	0.224	0.4916
85	0.21	0.4063
90	0.197	0.3823
95	0.187	0.3229
100	0.176	0.3125
150	0.114	0.1379

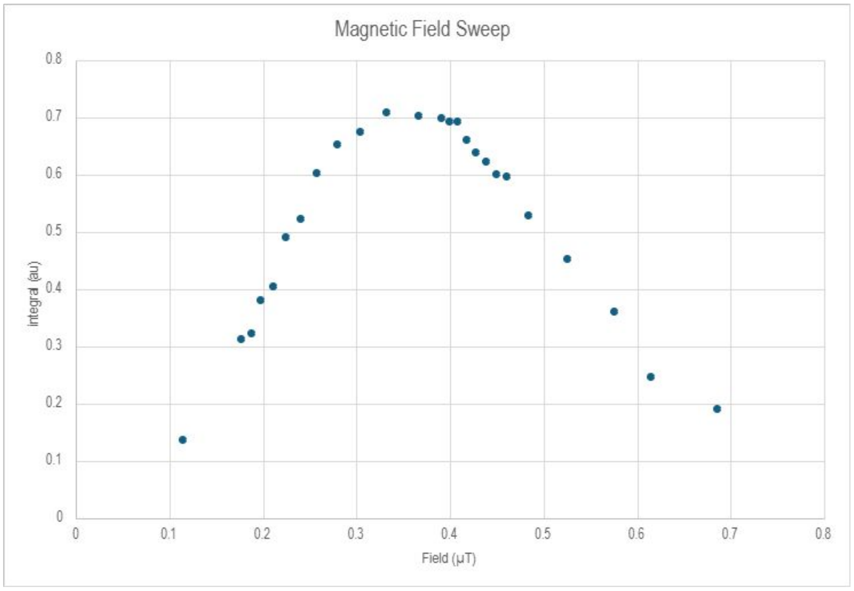

Figure S2. Magnetic field profile for [1-^{13}C]pyruvate in our hyperpolarizer setup.

2.4. Polarization Quantification

To calculate the polarization of the hyperpolarized signals, a reference spectrum of a known concentration and thermal polarization was acquired at 1.4 T using a pure sample of 17.18 M [1-^{13}C]acetic acid (**Figure S3**; Cambridge Isotope Laboratories, CLM-317). To calculate the thermal polarization of this sample, thermal Boltzmann statistics were used in Eq. S1, where γ is the gyromagnetic ratio (in this case $\gamma_{13C} = 67.23 \times 10^6\, s^{-1}T^{-1}$), B_0 is the magnetic field, $\hbar$ is Planck's constant, k_B is the Boltzmann constant, and T is the temperature.

$$P_{therm} = tanh\left(\frac{\gamma B_0 \hbar}{2k_B T}\right) \times 100 \qquad \textbf{Eq. S1}$$

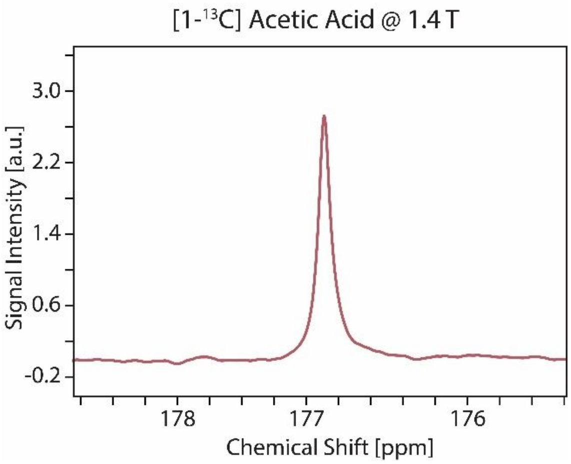

Figure S3. Reference spectrum used to calculate polarization. Single ^{13}C scan of pure [1-^{13}C]acetic acid (17.18 M) thermalized at 1.4 T using a benchtop NMR system (SpinSolve Carbon, Magritek, Ltd.).

Subsequently, this reference polarization can be used to calculate the polarization of the hyperpolarized signal using **Eq. S2**. Here, the statistical polarization of the reference signal (Ptherm) is modified by the ratios of the hyperpolarized signal integral and reference signal integral as well as the ratio of the concentrations. We assume that all *in situ* detected hyperpolarized signals have a concentration of 70 mM. To calculate the polarization of processed hyperpolarized samples with variable concentration, the actual concentration of the processed sample was used (see section 3.21 below).

$$P_{HP} = P_{therm} \times \frac{S_{HP}}{S_{REF}} \times \frac{C_{REF}}{C_{HP}} \quad \textbf{Eq. S2}$$

Representative sample calculation:

A representative detail of the polarization calculation from the main text **Table 1** is shown below:

$$P_{therm} = tanh\left(\frac{67.23 \times 10^{6}\, s^{-1}T^{-1} \times 1.4\, T \times 6.626 \times 10^{-34}\, Js^{-1}}{2 \times 1.38 \times 10^{-23} J\, K^{-1} \times 310\, K}\right) \times 100$$
$$= 1.16 \times 10^{-4}\, \%$$

NMR signal integral from thermally polarized reference ([1-^{13}C]acetic acid), S_{REF} = 24.71 a.u.

NMR signal integral from HP [1-^{13}C]pyruvate, S_{HP} = 7558.89 a.u.

Concentration of the thermally polarized acetic acid reference, C_{REF} = 17.18 M

Concentration of the HP [1-^{13}C]pyruvate sample, C_{HP} = 0.070 M

$$P_{HP} = 1.16 \times 10^{-4}\, \% \times \frac{7558.89}{24.71} \times \frac{17.18}{0.070} = 8.73\%$$

3. Experiment Details

3.1. Effective T_1 Relaxation Experiments

The relaxation of hyperpolarized [1-^{13}C]pyruvate was measured at various magnetic fields (0.4 µT, 50 µT, 1.1 mT, 5.8 mT, 48.5 mT, 200 mT, and 1.4 T) by several steps. Here, the fields are used are generated by the sources described in **Table S3** below.

Table S3. HP relaxation fields and field sources.

Relaxation Field	Field Source
0.4 µT	Polarization transfer field
50 µT	Earth's magnetic field
1.1 mT	Custom electromagnet
5.8 mT	Custom electromagnet
48.5 mT	Halbach array (Magritek)[7]
200 mT	Custom one-sided Halbach array
1.4 T	Detection field (SpinSolve Carbon, Magritek)

For each measurement, the sample was first polarized for 75 seconds at 0.4 μT with the hyperpolarization parameters as described in the method in Section 2.3 above. Subsequently, the sample was either kept at the same field (in the case of the 0.4 μT relaxation measurement) or moved to another magnetic field (in the case of the 50 μT, 1.1 mT, 5.8 mT, 48.5 mT, 200 mT, and 1.4 T magnetic field measurements). The sample was then allowed to relax for a delay time (shown in **Table S4** below). The sample was then transferred (in the case of all relaxation fields except 1.4 T, where the sample is already placed) to the 1.4 T benchtop NMR spectrometer. The signal remaining after the relaxation delay time was then acquired using a 7.0-degree flip angle, and the sample was then moved back to the polarizer and allowed to equilibrate temperature and magnetization for 75 seconds. The subsequent relaxation times were then all acquired in this fashion. Not all relaxation times were acquired for each magnetic field.

Table S4. Normalized HP signal integrals at each relaxation field.

Relaxation Delay Time [s]	1.4 T [a.u.]	200 mT [a.u.]	48.5 mT [a.u.]	5.8 mT [a.u]	1.1 mT [a.u.]	50 μT [a.u.]	0.4 μT [a.u.]
0	3473.02	2617.68	3256.43	3711.86	2166.90	2029.44	5788.15
2							5722.21
5							4991.08
8							4854.23
12							4523.16
15							3974.49
20							3563.99
25	2865.92	2025.95	2327.78	2086.14	1109.43	1042.62	2817.48
30							2373.24
40							1879.84
50	2931.00	1479.62	1690.06	1313.23	236.19	547.83	1416.69
60							1012.81
75	2318.29	1219.10	1194.75	818.64	346.00	271.25	652.79
100	1875.15	932.40	946.04	561.20	110.35	163.80	
125	1425.56	750.08	689.95	402.75	170.46	84.15	
150	1120.45	557.97	500.31	292.34	118.65	51.31	
175	876.40	447.61	389.83	209.67	80.10	29.32	
200	727.50						
225	511.47						

Data were fit using a mono-exponential decay function (**Eq. S3**). The resulting fits are shown in **Figure S4** and the fitting parameters are given in **Table S5**.

$$S = e^{-\frac{x}{T1}} \quad \textbf{Eq. S3}$$

Table S5. Fitting results for relaxation of HP [1-^{13}C]pyruvate.

Relaxation Field	Effective T_1 Value [s]
0.4 μT	35.1 ± 1.2
50 μT	38.1 ± 0.4
1.1 mT	33.0 ± 4.0
5.8 mT	50.7 ± 2.3
48.5 mT	79.2 ± 1.5
200 mT	97.4 ± 2.0
1.4 T	104.1 ± 1.6

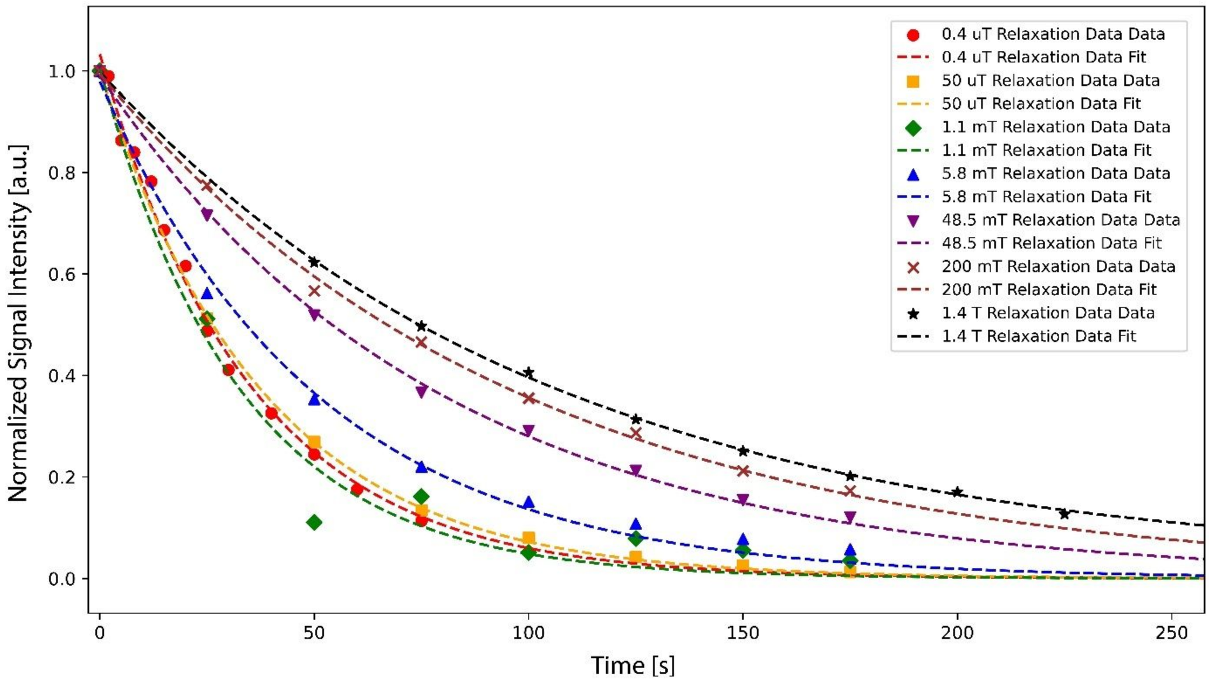

Figure S4. T_1 fits using a mono-exponential fit for the fields measured.

To ensure accurate characterization of the effective T_1 using the sample transfer method described above, we performed a parallel T_1 measurement of the T_1 at 1.4 T where the sample T_1 was measured using a series of 7-degree flip angles to sample the magnetization every 25 seconds. The comparison of this measurement and the prior 1.4 T T_1 measurement using the method described above is shown in **Figure S5**. The T_1 measurement is consistent across both methods, with a small lower deviation in the multi-pulse method as we do not account for magnetization consumption from the small tip angle (7-degree) pulse in our fitting protocol.

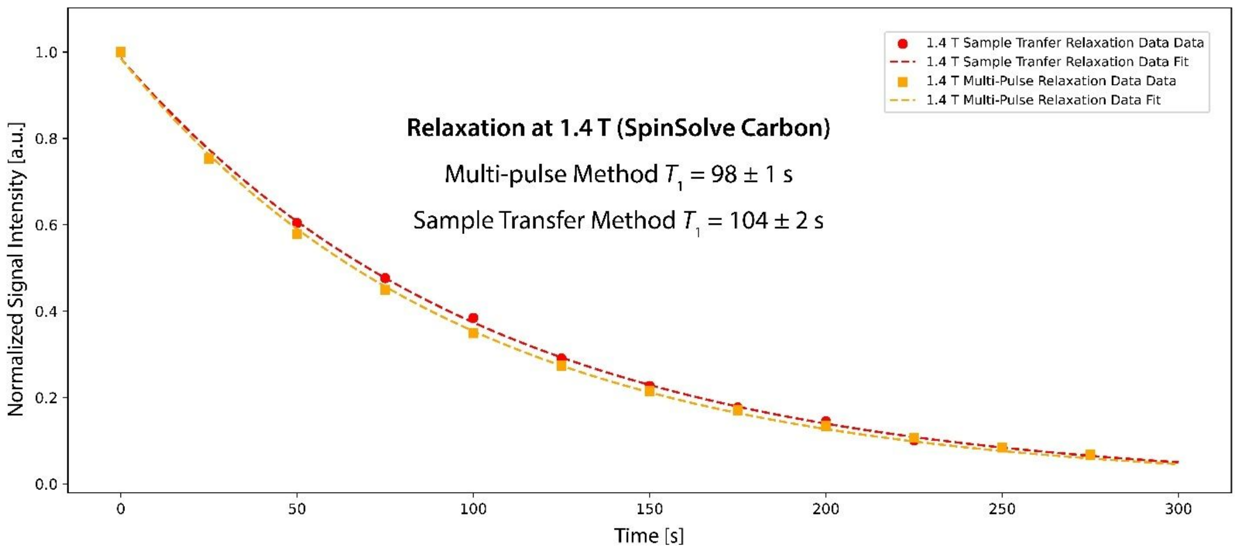

Figure S5. Comparison of the T_1 fits at 1.4 T using a multi-pulse sampling method and sample transfer method.

3.2. Hyperpolarized Solution Processing

To prepare biocompatible solutions of [1-^{13}C]pyruvate for cellular metabolic profiling experiments, the samples undergo additional purification steps to remove the majority of non-compatible components (acetone). The hyperpolarized sample is first transferred from the magnetic shields to a holding field (100 mT) and depressurized. The holding field is maintained throughout the entire purification process. The sample (initially containing acetone and water) is subjected to liquid-liquid extraction by combination with Sudan Blue II-dyed butyl acetate at a 4:1 volume ratio. The aqueous phase is then isolated via capillary extraction, diluted to approximately 700 μL, and pH-adjusted to 7.5 using Tris-EDTA buffer (40 mM Tris, 0.6 mM EDTA) while maintaining physiological osmolality at ~290 milliosmoles. For further purification, the buffered solution undergoes gas stripping in a heated Pyrex test tube (65-70°C) using nitrogen flow delivered at 500 sccm through a narrow capillary (1/16" outer diameter, 1/32" inner diameter). The final purified solution is collected in a 1 mL syringe for subsequent injection into NMR tubes for analysis.

3.2.1 Characterization of Processed Hyperpolarized [1-^{13}C]Pyruvate Solutions

Following the purification scheme outlined in **Fig. 2** and section 3.2 above, the concentrations of residual acetone and [1-^{13}C]pyruvate remaining in the solution were quantified by high-field NMR (500 MHz, Bruker) utilizing a calibration curve. The calibration curve for each compound was prepared via serial dilution of each compound, using 99.9% D_2O (Cambridge Isotope Laboratories, Inc.). Spectra for each sample was obtained at three concentrations for both acetone and pyruvate (10 mM, 50 mM, and 100 mM). For pyruvate, the ^{1}H signals resonance at 2.31 ppm was used and for acetone the ^{1}H resonance at 2.17 ppm was utilized. The resulting plots are shown in **Figure S6**, and the fits are shown in **Table S6**. Fits were calculated using linear regression in Excel using LINEST.

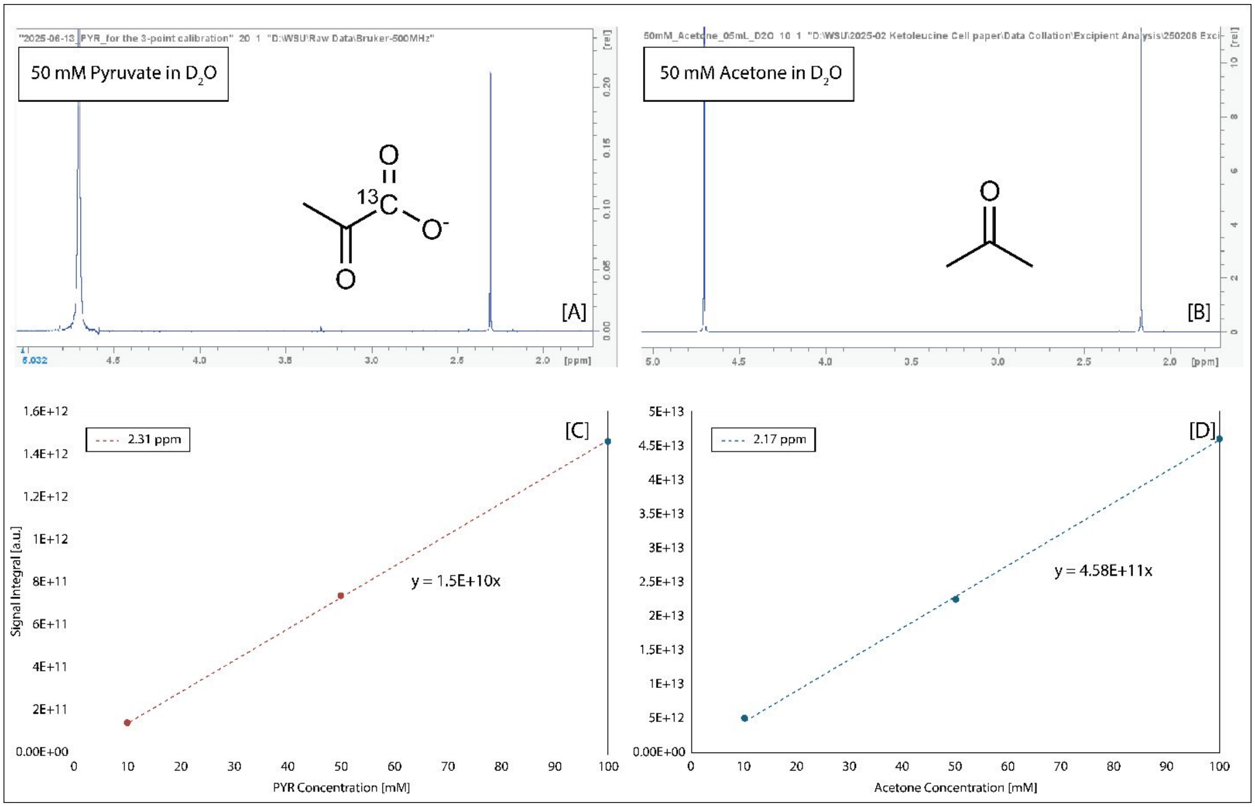

Figure S6. Reference spectra at 50 mM used for calculation of [A] pyruvate and [B] acetone concentrations. Fits for the determination of [C] pyruvate and [D] acetone concentrations.

Table S6. HP signal integrals at each relaxation field.

Concentration [mM]	Pyruvate Integrals [a.u]	Acetone Integrals [a.u.]
10	1.54E+11	5.09E+12
50	7.59E+11	2.25E+13
100	1.49E+12	4.59E+13

For quantification, a portion of each purified sample was analyzed using high-field NMR spectroscopy (500 MHz, Bruker), and the relevant peaks were integrated: 2.31 and 2.17 ppm. Peak integrals were compared to those of known standards to calculate concentrations. Full results are presented in **Table S7**.

Table S7. Concentration calculations using the corresponding fits from Table S4.

**Samples**		
Peak Position [ppm]	PYV (2.31)	Acetone (2.17)
**Sample 1**		
Sample 1 Data	1.17872E+12	1.30965E+13
Sample 1 Conc	78.63	28.62
Final Conc	*78.63*	*28.62*
**Sample 2**		
Sample 2 Data	9.9554E+11	1.01234E+13
Sample 2 Conc	66.41	22.12
Final Conc	*66.41*	*22.12*
**Sample 3**		
Sample 3 Data	1.14369E+12	1.18132E+13
Sample 3 Conc	76.29	25.81
Final Conc	*76.29*	*25.81*

3.3. Cellular Metabolic Profiling Experiments

For metabolic profiling experiments, yeast is first prepared and activated and then mixed with the hyperpolarized media. These steps are described in detail in the following sections:

3.3.1 Cell Preparation and Activation

Yeast cell suspensions (Saccharomyces cerevisiae, baker's yeast) were prepared by dispersing 7 g of yeast in 44 mL of phosphate buffer (0.2 M Na_2HPO_4/NaH_2PO_4, pH ~6.5) supplemented with 50 mM sucrose as a metabolic carbon source. The mixture was incubated at 35°C for a minimum of one hour to stimulate cellular activity, creating a viable cell population suitable for experimentation during hours 2-3 of the incubation period. The setup of the cell incubation is shown in **Figure S7**.

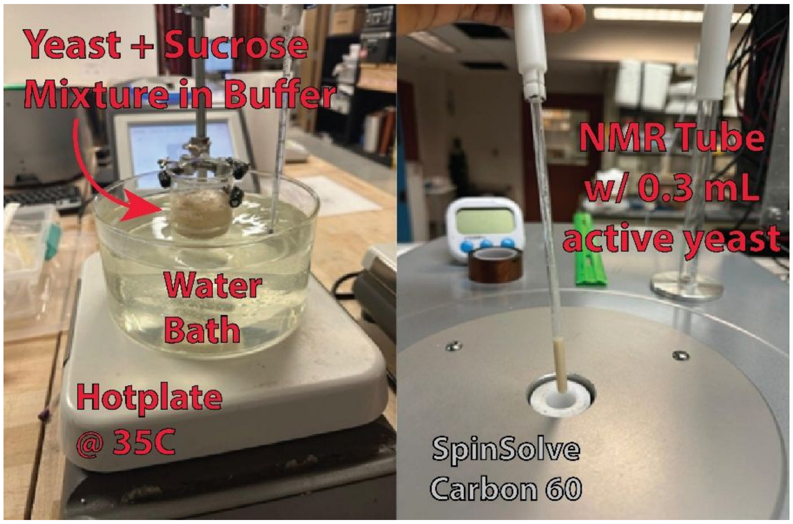

Figure S7. Experimental setup used for growth of *Saccharomyces cerevisiae* (Sc, baker's yeast) [LEFT] and loading of yeast in an NMR tube [RIGHT] before metabolic profiling experiments.

A 7g suspension of baker's yeast in 44mL buffer corresponds to about 3×10^9 cells per mL, so a 0.3mL aliquot initially contains roughly 1×10^9 cells. Under well-aerated, nutrient-rich conditions with a ~60-100 min doubling time, the population of a 0.3mL sample would increase to about 1.5–1.7×10^9 cells after one hour.[8] We estimate this to be approximately our concentration of yeast cells in our sample.

3.3.2 Metabolic Profiling Measurement Protocol

For hyperpolarized metabolic studies, 0.3 mL portions of activated cells were transferred into prewarmed 5 mm medium-wall NMR tubes and equilibrated in a 1.4 T benchtop NMR spectrometer (SpinSolve Carbon, Magritek) for 5 minutes before analysis. The cell suspension was gently mixed via capillary aspiration immediately prior to hyperpolarized substrate addition. The preparation of the cell mixture in an NMR tube is shown in **Figure S7**.

The purified hyperpolarized solution was then introduced at a 1:1 volume ratio through a narrow capillary (1/16" outer diameter, 1/32" inner diameter), followed by rapid mixing for approximately 5 seconds using the same capillary. The prepared sample was then positioned in the NMR spectrometer for real-time metabolic monitoring using a pseudo-2D ^{13}C acquisition sequence with optimized parameters: 7.0° excitation pulses, 1.6 s acquisition time, and 3.5 s repetition intervals.

4. Data Analysis and Processing of Cellular Metabolic Experiments

4.1. De-noising of Raw Signals with Singular Value Decomposition (SVD)

Singular value decomposition (SVD) provides an effective denoising approach by exploiting the low-rank characteristics of spectral signals within multidimensional data matrices. Hyperpolarized free induction decay (FID) signals are particularly amenable to matrix-based noise reduction methods, as their temporal compactness and spectral organization enable discrimination between genuine signal components and random noise. SVD denoising methodologies have gained widespread adoption in both *in vivo* and *in vitro* NMR applications where preserving quantitative accuracy of rapidly decaying signals is imperative.[9–11] A specialized SVD-based analysis workflow, adapted from established SVD NMR processing methods,[12] was developed to denoise and analyze complex FID signals from ^{13}C metabolic profile measurements. All computational procedures were executed in Python utilizing the *scipy* and *numpy* libraries.

Raw time-domain FID data underwent initial truncation to a standardized length ($2L-1$) before embedding into a square Hankel matrix with $L\times L$ dimensions. Matrix decomposition was performed using the LAPACK *gesdd* driver to optimize computational efficiency and numerical precision. For each dataset, only the principal k singular values were preserved, representing the most significant spectral modes. Matrix reconstruction followed by anti-diagonal averaging was then applied to maintain time-domain symmetry while attenuating uncorrelated noise. This truncation-reconstruction cycle was repeated (typically twice) to improve signal fidelity and consistency. Across complete datasets (metabolic profiling time series), the k parameter was iteratively decreased until the Fourier-transformed output satisfied quality assessment criteria.

Quality validation for individual spectra required: (1) absence of significant negative resonances; (2) signal confinement within ±1.5 ppm of anticipated metabolite chemical shifts; and (3) integrated peak intensities exceeding minimum thresholds. Resonances meeting these standards were selected for integration analysis. This strategy reduced manual intervention while ensuring reliable denoising across low signal-to-noise conditions, though occasionally resulted in missing

peaks within time series data. Post-SVD processing involved zero-filling to 65,536 data points followed by exponential apodization with a 1.5 Hz decay constant. Consistent phase correction was implemented across all samples using predetermined rotation parameters. The processed complex signal underwent Fourier transformation, baseline correction, and chemical shift calibration (ppm) based on experimental acquisition metadata.

Peak identification utilized local maximum detection above specified prominence and amplitude criteria in the real spectral component. Integration limits were established by symmetric expansion from peak centers until signal levels approached within 5% of baseline, estimated as the spectral median. Peak areas were computed using composite Simpson's integration rule. Both integrated intensities and peak amplitudes were recorded for subsequent modeling of metabolic profiles.

The code for this SVD de-noising protocol is provided separately.

This de-noised data for the metabolic flux analysis reported in the main text (Sample C1, **Figure 3 and Figure 4**) as well as the two additional data sets (Sample C2 and C3) included in the total analysis (**Table 2**) are given below in **Tables S8, S9, and S10**. This data is subsequently fit using the ordinary differential equation analysis described in section 4.2 below.

Table S8. SVD Denoised Integrals and Peak Heights, Sample C1

	Height	Height	Height	Height	Integral	Integral	Integral	Integral
Time	178.5	170	160	124	178.5	170	160	124
	hydrate	pyruvate	bicarbonate	CO2	hydrate	pyruvate	bicarbonate	CO2
0	166.45	6423.76	0.00	0.00	68.56	2973.57	0.00	0.00
3.5	229.43	7783.16	0.00	10.81	78.67	2946.22	0.00	3.28
7	404.26	11760.61	0.00	22.66	86.35	2890.28	0.00	4.95
10.5	509.32	13406.61	0.00	36.49	97.68	2816.27	0.00	6.61
14	591.25	14155.79	11.45	41.05	95.34	2990.83	6.46	7.59
17.5	664.05	14673.53	18.91	51.81	103.86	2890.48	5.44	8.35
21	744.11	15314.08	23.36	59.11	100.81	2790.76	5.46	8.79
24.5	804.72	15650.21	31.84	63.15	109.48	2718.10	6.86	9.04
28	839.96	15666.97	39.15	67.19	104.32	2379.82	6.62	9.44
31.5	884.47	15835.27	40.63	72.30	105.95	2307.34	5.83	9.57
35	906.30	15684.17	45.73	75.22	106.66	2243.95	6.27	9.41
38.5	940.78	15691.25	50.57	76.55	106.21	2172.34	6.32	9.64
42	943.63	15562.17	53.97	76.61	105.12	2109.74	7.14	9.50
45.5	974.32	15448.55	59.25	80.32	113.69	2038.21	6.99	9.40
49	971.82	15362.91	61.72	83.53	111.32	1987.80	6.71	9.14
52.5	981.71	15057.26	64.81	83.00	103.07	1919.78	6.98	9.63
56	999.03	14897.87	69.26	85.10	102.69	1863.78	8.12	9.45
59.5	998.64	14637.17	67.82	82.92	101.74	1802.44	7.53	9.05
63	998.47	14329.52	67.81	82.79	107.10	1741.18	7.38	9.17
66.5	964.09	14374.00	74.31	82.98	96.52	1713.13	7.56	9.21
70	963.51	13792.49	73.29	86.24	95.09	1624.72	7.16	9.27
73.5	966.00	13730.32	77.10	85.25	101.08	1585.67	7.07	8.90
77	945.45	13387.24	75.94	78.80	91.44	1534.77	7.30	8.26

80.5	929.58	13374.36	82.77	81.75	88.62	1507.81	7.81	8.26
84	907.28	12872.59	74.42	80.26	86.81	1446.79	6.95	8.12
87.5	899.89	12599.40	75.98	77.14	84.71	1462.44	7.68	7.98
91	889.28	12269.24	76.33	76.97	83.30	1413.24	7.58	7.80
94.5	859.30	11787.09	76.89	78.64	81.28	1301.49	7.91	9.28
98	843.61	11743.22	80.59	75.99	80.01	1282.16	7.37	8.11
101.5	817.52	11122.49	81.06	75.44	77.98	1222.95	9.68	9.02
105	801.68	11035.36	78.54	76.85	76.11	1260.74	8.87	9.26
108.5	783.19	10507.22	80.85	74.69	73.66	1379.44	13.07	9.15
112	760.24	10241.07	73.19	76.06	71.43	1106.05	8.82	9.12
115.5	740.27	9962.62	72.52	70.70	69.87	1074.32	8.88	8.17
119	721.73	9949.25	77.66	68.35	68.13	1105.70	8.49	8.19
122.5	706.25	9445.27	71.27	69.88	65.93	1005.07	7.47	8.29
126	691.10	9269.27	72.66	66.27	63.91	979.61	8.27	8.11
129.5	672.23	9151.59	68.22	66.54	62.28	1003.42	6.95	7.44
133	655.77	8700.27	67.40	61.62	60.88	909.42	7.31	7.43
136.5	636.16	8484.06	63.73	62.80	58.60	882.33	6.71	7.48
140	622.54	8554.53	70.69	56.05	64.62	915.52	7.10	6.60
143.5	609.18	8281.94	62.31	55.46	55.80	1011.96	10.13	6.28
147	587.64	7972.12	61.60	54.18	53.64	849.78	7.27	6.11
150.5	566.36	7760.74	60.96	51.54	51.90	825.82	6.52	5.54
154	546.79	7355.09	61.39	54.10	50.35	750.80	6.47	15.45
157.5	535.10	7305.89	61.91	6.84	48.26	772.96	6.48	9.76
161	520.15	6884.00	63.49	47.25	47.29	697.62	6.45	5.45
164.5	507.44	6726.14	51.48	48.97	45.71	678.90	6.04	5.85
168	490.36	6668.02	57.18	47.33	44.28	666.39	6.05	5.43
171.5	473.73	6384.91	49.53	47.94	42.77	638.80	5.71	5.41
175	456.87	6230.78	49.89	43.71	41.57	621.86	4.95	5.02
178.5	444.53	6014.19	49.15	37.53	39.95	600.84	5.57	4.33
182	433.43	5861.18	47.62	38.42	39.29	582.88	5.09	4.15
185.5	415.31	5637.03	46.51	37.51	37.72	562.26	54.12	4.51
189	401.82	5410.86	50.75	39.08	37.34	612.24	6.73	4.38
192.5	386.19	5249.38	45.83	36.90	34.83	521.90	5.20	4.28
196	382.35	5023.69	48.55	35.79	34.84	502.98	4.53	4.33
199.5	368.80	4906.34	36.94	34.85	33.63	488.00	4.03	4.13
203	353.46	4840.70	39.26	32.82	32.20	478.44	4.09	3.63
206.5	334.02	4636.38	36.85	32.78	30.62	458.08	4.11	3.29
210	339.91	4508.72	40.12	31.36	30.52	445.14	4.40	3.67
213.5	327.17	4388.67	33.79	32.21	29.29	431.88	3.94	3.63
217	311.69	4232.30	36.69	29.60	28.05	417.18	3.85	3.68
220.5	302.09	4035.35	38.56	29.05	27.54	399.70	3.90	3.23
224	290.15	3952.98	30.81	28.81	26.43	388.23	3.37	3.12
227.5	287.62	3808.18	32.61	23.71	26.16	375.65	3.81	3.22
231	277.33	3748.47	31.64	24.24	25.03	366.19	3.59	2.81

234.5	258.60	3635.82	29.69	23.92	23.30	355.94	3.82	2.72
238	258.38	3529.34	29.28	23.54	23.90	343.64	3.17	2.78
241.5	249.83	3414.80	25.85	19.42	23.12	332.67	3.62	2.18
245	238.33	3263.42	26.75	21.63	21.48	320.26	3.29	2.51
248.5	236.20	3165.41	27.36	22.85	21.46	308.93	3.42	2.63
252	222.28	3113.40	24.59	20.63	18.99	301.63	3.09	2.12
255.5	217.58	3014.07	24.80	20.26	19.85	292.58	2.59	2.54
259	208.88	2857.92	25.35	18.31	17.94	279.23	2.85	2.10
262.5	198.08	2822.91	23.49	19.02	17.04	273.27	2.84	2.30
266	194.84	2720.55	21.16	17.25	17.05	265.80	3.13	2.19
269.5	186.80	2630.88	17.85	15.35	17.84	256.32	2.51	1.88
273	183.06	2571.23	22.75	19.20	16.44	249.98	2.59	2.15
276.5	175.71	2451.25	21.09	17.85	15.16	239.13	2.45	2.27
280	170.54	2386.17	16.04	15.66	14.44	232.49	1.92	1.97
283.5	169.13	2288.00	19.82	16.96	14.47	224.91	2.31	1.56
287	162.80	2223.29	17.64	16.14	13.67	218.33	1.96	1.75
290.5	154.91	2158.00	18.86	13.84	13.27	212.84	2.26	1.33
294	150.30	2093.51	17.08	15.11	12.77	204.82	2.14	2.13
297.5	148.54	2046.16	17.97	13.80	12.60	198.31	2.17	1.92
301	141.91	1997.86	13.56	11.47	12.29	193.73	1.86	1.32
304.5	141.22	1909.14	18.80	10.75	11.72	186.70	1.99	1.17
308	138.21	1884.32	15.34	12.13	11.80	182.36	1.57	1.97
311.5	126.34	1801.07	18.33	11.75	10.66	174.85	1.83	1.34
315	129.18	1747.18	13.89	13.27	11.04	170.87	1.34	1.86
318.5	124.97	1687.63	13.50	11.33	10.62	163.47	1.86	1.52
322	116.36	1631.22	11.89	12.01	9.83	158.66	1.47	1.72
325.5	119.14	1606.90	13.37	10.43	10.39	155.89	1.63	1.88
329	114.64	1547.48	12.90	9.30	9.97	150.28	1.48	1.33
332.5	105.41	1500.67	13.53	9.85	9.51	144.82	1.82	1.10
336	104.71	1459.08	13.69	0.00	8.92	140.85	1.69	0.00
339.5	101.51	1394.52	10.62	0.00	8.54	135.38	1.45	0.00
343	98.59	1389.47	10.37	8.93	8.33	133.94	1.37	0.82
346.5	94.53	1366.72	11.82	9.55	7.93	130.43	1.32	1.07
350	90.45	1332.82	11.09	0.00	7.62	125.87	1.12	0.00
353.5	94.55	1307.47	10.26	0.00	7.91	122.83	0.81	0.00
357	88.70	1252.56	8.73	6.86	7.22	118.93	0.85	1.71
360.5	85.60	1214.49	10.20	0.00	6.97	114.23	1.26	0.00
364	83.65	1180.00	0.00	0.00	6.85	110.82	0.00	0.00
367.5	80.19	1150.08	9.89	8.75	6.67	109.36	1.01	0.75
371	82.76	1110.65	12.11	0.00	6.74	104.88	1.13	0.00
374.5	79.29	1075.49	7.27	0.00	6.62	102.24	0.67	0.00
378	76.33	1065.23	9.04	0.00	6.32	100.35	0.87	0.00
381.5	73.02	1031.19	10.26	10.61	5.99	97.18	1.69	1.09
385	73.39	1000.21	10.41	0.00	5.85	94.28	1.09	0.00

388.5	69.91	981.61	0.00	0.00	5.80	92.58	0.00	0.00
392	67.38	942.09	0.00	0.00	5.64	88.71	0.00	0.00
395.5	71.76	916.58	0.00	0.00	5.69	85.47	0.00	0.00
399	63.33	887.01	0.00	0.00	5.04	83.47	0.00	0.00
402.5	59.56	857.13	0.00	0.00	4.80	80.79	0.00	0.00
406	59.66	841.28	0.00	0.00	5.12	78.63	0.00	0.00
409.5	55.98	816.54	0.00	0.00	4.78	75.70	0.00	0.00
413	55.50	797.11	0.00	0.00	4.84	74.92	0.00	0.00
416.5	54.29	776.53	7.96	0.00	4.53	72.48	0.76	0.00
420	55.63	746.94	6.85	0.00	4.38	69.75	0.52	0.00
423.5	51.58	739.11	0.00	0.00	4.23	68.37	0.00	0.00
427	49.73	713.46	0.00	0.00	4.21	66.17	0.00	0.00
430.5	48.54	691.13	0.00	0.00	3.97	63.83	0.00	0.00
434	54.02	673.56	7.78	0.00	4.35	62.44	0.66	0.00
437.5	44.10	652.99	0.00	0.00	3.58	61.16	0.00	0.00
441	44.30	630.55	0.00	0.00	3.43	58.75	0.00	0.00
444.5	42.99	604.78	0.00	0.00	3.63	56.78	0.00	0.00
448	46.41	598.22	0.00	0.00	3.63	55.25	0.00	0.00
451.5	40.19	579.08	7.64	0.00	3.18	54.29	0.56	0.00
455	41.43	559.50	0.00	0.00	3.20	52.79	0.00	0.00
458.5	36.60	540.29	0.00	0.00	2.88	50.22	0.00	0.00
462	41.98	527.96	0.00	0.00	3.45	49.46	0.00	0.00
465.5	39.24	508.50	0.00	0.00	3.23	47.99	0.00	0.00
469	37.58	497.70	0.00	0.00	3.04	46.93	0.00	0.00
472.5	36.71	484.16	0.00	0.00	3.10	45.04	0.00	0.00
476	36.43	472.39	0.00	0.00	2.94	44.02	0.00	0.00
479.5	32.74	462.07	0.00	0.00	2.42	43.06	0.00	0.00
483	32.09	446.94	0.00	0.00	2.56	41.31	0.00	0.00
486.5	27.79	436.50	0.00	0.00	2.46	40.55	0.00	0.00
490	28.83	425.02	0.00	0.00	2.35	39.41	0.00	0.00
493.5	28.58	409.36	0.00	0.00	2.50	37.96	0.00	0.00
497	31.00	400.55	0.00	0.00	2.68	37.61	0.00	0.00
500.5	30.74	381.65	0.00	0.00	2.32	35.26	0.00	0.00
504	30.19	373.52	0.00	0.00	2.47	34.71	0.00	0.00
507.5	25.85	360.57	0.00	0.00	2.23	34.00	0.00	0.00
511	23.50	357.58	0.00	0.00	1.78	33.32	0.00	0.00
514.5	21.71	343.23	0.00	0.00	2.06	32.15	0.00	0.00
518	22.29	333.80	0.00	0.00	1.82	30.80	0.00	0.00
521.5	21.09	324.05	0.00	0.00	1.69	30.13	0.00	0.00

Table S9. SVD Denoised Integrals and Peak Heights, Sample C2

	Height	Height	Height	Height	Integral	Integral	Integral	Integral
Time	178.5	170	160	124	178.5	170	160	124
	hydrate	pyruvate	bicarbonate	CO2	hydrate	pyruvate	bicarbonate	CO2

0	276.74	10622.05	0.00	0.00	72.30	3063.47	0.00	0.00
3.5	408.58	15098.82	0.00	15.96	75.21	3011.78	0.00	2.84
7	457.91	16373.94	0.00	24.17	78.99	3001.41	0.00	4.60
10.5	479.91	16613.98	10.41	33.47	83.50	3192.79	3.27	6.45
14	490.57	16430.24	15.43	40.54	82.51	2865.69	3.41	7.51
17.5	502.96	16310.37	21.75	45.90	86.79	2793.82	4.70	8.29
21	506.41	15967.30	27.18	49.34	83.79	2978.31	6.55	8.79
24.5	510.07	15677.94	30.92	50.18	84.38	2657.59	6.28	9.75
28	512.12	15415.06	38.69	53.50	84.48	2596.07	8.02	9.71
31.5	512.46	15186.63	44.12	55.78	84.74	2550.49	9.04	10.73
35	513.99	14945.74	49.23	57.99	84.37	2491.33	10.13	10.04
38.5	509.68	14492.44	51.04	59.98	84.50	2420.72	10.40	10.63
42	510.59	14257.36	56.83	56.42	84.63	2357.87	11.26	9.08
45.5	507.85	13984.70	60.35	56.25	83.19	2316.12	10.56	9.62
49	503.40	13537.05	63.73	58.38	82.77	2250.88	12.30	10.64
52.5	500.27	13350.36	66.12	59.55	82.85	2203.11	11.01	10.44
56	497.48	13083.01	69.20	55.34	81.43	2147.05	12.01	9.12
59.5	490.63	12658.80	70.50	57.02	81.11	2095.66	12.70	10.60
63	488.67	12401.06	71.97	59.01	80.90	2047.47	12.09	10.89
66.5	483.91	12232.85	75.38	56.12	80.07	2003.11	13.38	9.86
70	475.43	11931.49	75.10	57.40	78.65	1950.52	12.88	9.31
73.5	472.78	11549.83	81.79	57.63	77.94	1893.31	14.29	10.64
77	468.05	11380.58	81.42	52.93	77.36	1860.40	14.32	9.00
80.5	459.54	11087.96	80.06	53.95	75.52	1810.12	13.60	10.04
84	456.26	10884.21	80.66	54.12	75.33	1773.56	14.27	9.96
87.5	447.10	10556.57	86.03	52.43	74.64	1718.30	13.92	10.06
91	443.15	10314.65	83.56	53.06	73.17	1681.47	15.12	9.95
94.5	439.10	10099.08	83.81	53.26	71.87	1641.39	14.76	9.61
98	431.29	9883.21	86.04	53.75	71.17	1605.56	14.55	10.08
101.5	425.18	9694.58	83.19	52.02	69.92	1575.31	14.71	9.75
105	418.15	9400.80	87.92	48.10	69.17	1530.25	14.38	9.22
108.5	411.29	9270.38	85.77	48.92	67.96	1501.57	15.11	9.32
112	405.99	9082.39	85.76	49.01	67.54	1469.06	14.34	8.71
115.5	395.60	8841.74	83.71	48.76	65.87	1431.24	14.74	9.23
119	392.64	8630.16	84.20	43.47	65.08	1396.81	14.49	8.11
122.5	387.21	8358.17	89.12	51.12	65.08	1367.33	16.06	11.74
126	385.27	8214.50	90.85	51.91	64.71	1339.39	17.40	12.29
129.5	379.12	8073.09	89.32	49.07	64.03	1303.77	16.80	10.65
133	370.35	7910.14	86.74	48.89	62.09	1279.34	16.17	11.64
136.5	367.33	7685.37	90.30	46.22	61.41	1241.35	16.55	11.29
140	361.26	7531.36	88.82	46.69	61.07	1214.58	16.71	11.01
143.5	351.32	7356.43	84.40	44.95	59.01	1189.21	15.99	10.78
147	351.58	7275.21	84.51	42.60	59.05	1168.08	16.11	10.14
150.5	343.49	7066.27	85.36	45.68	57.22	1131.24	16.18	10.12

154	339.22	6897.90	81.71	45.14	57.64	1106.66	14.36	9.78
157.5	333.19	6742.82	82.92	40.10	55.72	1083.18	14.78	9.87
161	326.00	6662.00	82.15	41.50	54.73	1064.37	15.39	9.78
164.5	319.35	6429.97	80.96	38.64	53.56	1037.75	14.37	8.86
168	317.29	6299.36	78.21	38.27	52.68	1016.51	15.33	8.98
171.5	310.58	6185.35	75.93	39.59	52.03	1074.02	16.75	8.59
175	304.45	6078.24	78.40	37.20	50.58	971.53	14.52	8.13
178.5	297.45	5919.76	75.71	35.81	50.14	951.37	13.92	8.78
182	293.04	5834.01	73.22	36.99	49.50	934.23	13.33	8.38
185.5	289.19	5703.37	73.54	35.38	48.43	907.68	13.60	7.61
189	285.01	5624.42	73.59	36.22	47.82	888.12	12.68	7.78
192.5	280.45	5543.77	71.30	32.85	45.98	876.72	12.19	7.46
196	275.03	5377.24	70.41	34.79	45.53	850.63	12.99	7.14
199.5	269.10	5311.67	69.94	32.86	44.85	835.44	12.02	7.25
203	266.33	5184.48	67.37	30.67	44.51	816.10	12.69	7.79
206.5	261.52	5073.09	65.94	32.77	43.40	802.27	12.52	7.35
210	253.97	4974.58	64.61	30.73	42.54	786.17	12.08	6.60
213.5	251.67	4875.36	63.50	31.81	41.79	769.42	12.11	7.81
217	247.32	4765.68	66.05	29.90	41.33	753.97	11.49	6.94
220.5	240.63	4662.74	65.85	28.04	39.74	737.18	11.74	6.95
224	236.93	4544.45	63.22	30.13	39.09	788.68	14.63	6.93
227.5	234.26	4459.49	59.58	27.23	38.39	705.72	11.32	6.77
231	230.84	4429.77	64.94	26.35	38.58	693.69	11.63	7.17
234.5	223.53	4336.11	58.05	27.61	36.82	677.45	10.41	6.92
238	220.44	4269.49	60.26	27.16	35.46	663.70	12.02	6.21
241.5	217.83	4154.12	57.20	26.45	35.89	646.46	12.15	5.56
245	216.67	4053.40	57.97	24.98	35.13	634.63	9.86	6.02
248.5	211.61	4007.57	0.00	23.09	34.32	622.08	0.00	5.32
252	209.20	3945.16	54.64	23.19	33.84	611.92	9.48	5.76
255.5	207.24	3811.74	53.83	24.02	33.32	594.14	9.68	6.20
259	202.22	3822.08	54.54	24.19	33.12	584.40	9.51	5.38
262.5	198.33	3727.21	52.64	25.00	32.28	575.39	9.70	5.84
266	198.84	3638.98	48.36	19.56	32.22	562.96	9.46	4.43
269.5	194.88	3631.25	51.39	22.06	31.63	549.08	8.51	5.46
273	188.31	3535.18	49.81	22.95	30.28	536.54	8.81	5.29
276.5	186.30	3466.81	49.09	19.97	30.68	525.59	10.89	4.89
280	183.32	3370.45	49.17	17.93	29.48	516.97	8.67	4.35
283.5	180.71	3335.81	48.57	21.25	28.51	506.31	9.10	5.15
287	178.99	3253.72	46.46	19.20	28.20	493.00	8.02	4.60
290.5	177.80	3171.55	46.49	18.69	27.82	484.82	9.53	4.84
294	172.57	3127.29	48.44	20.61	28.15	473.68	9.82	4.66
297.5	167.13	3055.94	44.21	19.72	26.25	463.74	9.69	4.42
301	164.50	2987.10	44.12	19.67	26.35	453.18	9.64	3.86
304.5	160.83	2919.02	43.45	17.89	25.33	443.08	7.59	4.47

308	158.75	2872.69	41.55	6.94	24.44	438.71	6.91	3.89
311.5	154.11	2801.92	40.37	18.53	24.40	425.35	6.62	4.34
315	153.70	2735.02	41.86	15.56	23.57	415.26	8.60	3.62
318.5	146.75	2707.37	40.51	17.39	22.92	406.83	8.75	4.16
322	145.48	2653.11	40.37	15.66	22.83	398.78	8.33	3.58
325.5	142.76	2602.88	37.68	14.68	21.77	391.32	8.05	3.95
329	141.43	2534.04	38.20	16.37	21.62	384.76	8.27	4.94
332.5	139.57	2515.31	37.18	13.40	21.07	376.16	7.09	3.30
336	136.11	2460.75	37.76	13.88	20.62	369.38	7.87	3.81
339.5	135.24	2405.57	35.52	13.85	21.13	362.33	7.24	4.03
343	131.14	2364.12	35.61	15.86	20.54	355.06	7.57	3.26
346.5	128.46	2315.09	35.60	12.66	19.94	346.51	7.33	3.68
350	127.29	2286.12	35.30	14.09	18.94	339.15	7.02	2.80
353.5	123.31	2248.37	33.06	12.19	19.10	333.49	5.49	3.82
357	121.68	2197.08	32.01	13.56	18.72	327.63	6.51	3.34
360.5	119.50	2157.34	33.27	12.92	18.67	320.68	7.06	2.85
364	119.15	2154.72	31.51	10.30	17.97	316.61	6.80	2.85
367.5	116.52	2100.45	31.44	12.92	17.62	307.69	6.23	3.20
371	113.77	2054.23	29.44	9.81	17.37	304.69	5.99	2.95
374.5	115.36	2016.27	29.70	10.05	17.71	299.02	8.42	2.78
378	109.63	1968.40	28.50	13.90	17.13	328.89	9.52	2.99
381.5	107.13	1940.02	29.74	12.99	17.00	286.38	5.65	2.84
385	107.32	1905.94	28.06	12.23	16.47	282.21	5.94	2.87
388.5	104.52	1861.25	26.18	11.01	16.42	276.21	5.48	3.51
392	102.41	1835.01	27.84	0.00	16.30	271.32	5.62	0.00
395.5	100.77	1796.98	27.17	9.96	15.24	264.07	5.49	2.79
399	97.63	1759.89	28.53	14.04	15.20	258.54	5.78	2.51
402.5	100.54	1732.04	27.61	10.77	15.22	253.81	4.74	1.95
406	96.80	1707.63	24.34	11.05	15.11	249.40	5.32	2.66
409.5	93.13	1680.63	25.42	10.61	14.09	244.08	4.55	2.16
413	91.87	1639.77	25.34	0.00	14.09	236.31	4.74	0.00
416.5	90.87	1641.74	22.51	0.00	13.57	235.70	4.66	0.00
420	87.93	1581.77	22.77	0.00	13.67	229.62	4.50	0.00
423.5	87.73	1560.18	23.38	0.00	13.37	224.91	4.76	0.00
427	86.05	1521.07	22.03	9.12	13.84	219.68	4.56	1.80
430.5	86.53	1501.77	20.91	0.00	12.73	216.30	4.49	0.00
434	82.02	1457.57	21.98	0.00	12.02	212.00	4.70	0.00
437.5	79.19	1425.58	22.51	0.00	11.59	206.71	4.18	0.00
441	79.60	1404.97	20.90	0.00	11.59	202.71	4.05	0.00
444.5	79.87	1395.30	18.82	9.43	11.85	198.70	3.97	1.78
448	77.03	1370.63	21.08	0.00	11.22	196.04	4.00	0.00
451.5	75.58	1334.02	19.57	0.00	11.09	191.27	4.04	0.00
455	75.41	1301.33	21.58	5.38	11.67	185.90	4.27	3.15
458.5	74.25	1302.32	20.71	0.00	11.14	184.04	3.77	0.00

462	72.54	1261.17	16.78	0.00	10.93	180.19	4.01	0.00
465.5	70.83	1233.85	18.68	0.00	10.70	176.53	3.65	0.00
469	70.29	1218.20	16.79	10.16	10.52	171.46	3.55	1.30
472.5	67.22	1207.37	17.67	0.00	10.02	172.70	3.73	0.00
476	65.17	1187.26	13.42	0.00	10.06	167.24	2.89	0.00
479.5	65.25	1159.14	17.69	0.00	10.12	162.85	3.16	0.00
483	62.33	1107.82	15.40	0.00	9.56	157.28	3.61	0.00
486.5	63.91	1120.32	17.63	0.00	9.27	158.33	3.09	0.00
490	57.93	1097.90	14.72	0.00	8.93	155.82	2.59	0.00
493.5	61.62	1057.09	15.40	0.00	9.48	149.97	3.36	0.00
497	57.77	1035.54	15.83	0.00	8.27	148.23	2.91	0.00
500.5	57.54	1015.37	14.98	0.00	8.71	142.46	3.03	0.00
504	55.78	1010.14	12.75	0.00	8.52	142.18	2.90	0.00
507.5	54.57	983.38	12.00	0.00	8.19	139.23	2.82	0.00
511	55.53	965.48	12.78	0.00	8.73	135.11	2.60	0.00
514.5	53.36	938.42	12.29	0.00	7.75	134.07	3.26	0.00
518	52.64	909.34	15.42	0.00	7.89	129.23	3.17	0.00
521.5	52.88	907.33	15.03	5.17	8.29	127.39	2.51	1.15

Table S10. SVD Denoised Integrals and Peak Heights, Sample C3

Time	Height 178.5 hydrate	Height 170 pyruvate	Height 160 bicarbonate	Height 124 CO2	Integral 178.5 hydrate	Integral 170 pyruvate	Integral 160 bicarbonate	Integral 124 CO2
0	375.30	12248.15	0.00	0.00	66.92	2639.35	0.00	0.00
3.5	533.07	16059.37	0.00	21.26	69.54	2577.15	0.00	3.19
7	632.90	17833.03	11.06	38.59	72.98	2810.97	4.32	4.57
10.5	699.17	18651.40	19.67	46.20	75.07	2777.73	11.40	5.86
14	671.29	17965.60	24.72	53.88	73.83	2713.26	12.39	6.34
17.5	674.16	17495.87	30.63	56.65	74.92	2623.34	9.48	6.93
21	665.18	17070.05	39.21	60.47	74.10	2538.78	7.13	7.96
24.5	685.93	17398.59	42.90	67.23	79.38	2214.34	5.48	8.01
28	722.38	17868.44	57.44	71.96	73.77	2572.62	9.28	8.11
31.5	789.30	17466.62	66.52	74.30	75.96	2165.45	7.14	8.31
35	825.88	0.00	83.87	80.67	80.96	0.00	7.92	8.08
38.5	850.92	0.00	85.76	86.41	74.17	0.00	10.03	8.49
42	862.36	0.00	97.32	87.44	73.87	0.00	9.24	8.25
45.5	864.76	0.00	118.64	90.39	73.82	0.00	11.27	8.85
49	848.41	0.00	117.28	85.70	72.96	0.00	11.47	8.78
52.5	827.88	0.00	112.70	90.10	72.88	0.00	10.68	9.22
56	810.66	16456.46	118.56	83.30	72.63	1815.22	12.46	8.77
59.5	792.10	15589.15	123.06	83.26	71.32	1745.08	11.79	8.67
63	779.02	0.00	124.05	85.18	70.70	0.00	11.78	8.50
66.5	772.59	14736.92	135.91	79.22	70.10	1658.09	12.46	8.12
70	759.29	14721.19	126.87	80.57	67.97	1647.01	12.26	8.42

73.5	736.24	14175.35	145.17	79.30	67.78	1588.93	13.79	8.75
77	716.96	0.00	129.02	75.10	65.88	0.00	13.29	7.97
80.5	690.14	0.00	135.06	76.55	63.86	0.00	13.55	8.45
84	670.64	12707.75	0.00	74.53	63.29	1459.55	0.00	8.15
87.5	647.73	12298.74	131.84	71.00	61.85	1424.24	13.51	7.65
91	633.37	12117.32	148.91	71.90	64.95	1411.23	16.41	9.45
94.5	611.19	11029.70	133.97	69.99	62.26	1333.57	15.29	8.86
98	593.32	10723.17	141.13	67.72	61.12	1243.31	15.42	8.82
101.5	571.41	10598.51	124.07	70.13	59.82	1283.74	16.27	9.11
105	551.45	10199.50	123.88	65.67	59.97	1197.28	15.50	9.08
108.5	514.27	9494.60	120.17	57.56	57.87	1159.56	14.70	8.68
112	503.56	9454.64	122.96	59.48	57.59	1152.48	15.74	8.72
115.5	474.50	9116.81	113.62	58.12	55.09	1123.54	14.22	8.95
119	459.49	8460.85	110.87	54.00	54.51	1079.14	14.18	8.38
122.5	455.55	8288.55	113.62	53.77	52.89	1057.45	22.68	8.54
126	458.44	8087.61	113.35	57.14	51.89	1025.05	14.80	8.24
129.5	460.58	8067.84	116.80	55.18	51.25	1010.33	14.94	8.27
133	466.71	8094.60	121.54	55.50	49.39	985.09	14.17	7.47
136.5	466.69	7861.75	117.53	54.44	47.47	950.51	13.31	7.41
140	485.42	8165.13	122.91	53.21	48.16	931.84	12.96	6.74
143.5	485.62	8071.45	128.05	53.22	46.15	934.74	13.07	6.73
147	503.03	8296.60	132.39	52.04	45.83	908.65	12.27	5.78
150.5	513.08	0.00	130.58	55.78	45.23	0.00	12.53	6.54
154	518.82	0.00	135.18	55.76	44.81	0.00	13.11	6.21
157.5	517.55	0.00	136.66	54.79	44.29	0.00	18.05	5.97
161	502.33	0.00	135.49	53.93	43.67	0.00	18.90	6.20
164.5	482.90	0.00	127.84	48.31	42.37	0.00	12.41	5.89
168	476.40	0.00	128.81	52.35	41.95	0.00	18.70	5.81
171.5	464.95	0.00	123.09	51.52	41.22	0.00	11.58	5.46
175	455.67	0.00	118.62	49.59	40.12	0.00	11.13	5.43
178.5	446.04	0.00	114.70	48.12	39.25	0.00	10.69	5.45
182	435.30	0.00	117.74	46.75	38.40	0.00	10.87	5.15
185.5	427.68	0.00	115.84	43.92	37.42	0.00	10.58	5.19
189	416.77	0.00	107.21	43.24	36.62	0.00	10.32	4.46
192.5	403.85	0.00	104.68	41.84	35.73	0.00	10.02	4.64
196	393.25	0.00	103.19	38.06	34.48	0.00	9.77	4.42
199.5	375.12	6380.40	104.59	38.84	33.42	631.81	10.00	4.55
203	385.37	6233.77	101.74	40.14	33.44	617.68	9.14	4.51
206.5	369.70	6128.09	100.05	39.05	32.32	595.35	9.16	4.33
210	362.45	5994.20	103.65	36.98	31.69	561.97	9.68	4.14
213.5	366.86	0.00	98.36	35.79	32.06	0.00	8.89	4.16
217	354.80	5764.83	93.52	34.07	30.90	554.73	8.47	3.95
220.5	351.04	5579.08	91.20	32.50	30.88	538.11	8.40	3.78
224	339.26	5505.88	92.62	34.85	29.66	528.54	8.53	3.90

227.5	334.13	5371.10	91.42	31.22	29.37	514.70	8.44	3.73
231	320.13	5253.98	87.71	34.02	27.70	501.37	8.14	3.79
234.5	317.57	5144.64	89.50	31.44	27.26	491.17	8.10	3.66
238	317.81	5077.95	84.54	26.86	27.71	481.13	7.93	3.01
241.5	297.53	4947.94	81.49	32.06	24.85	474.53	7.37	3.37
245	296.00	4846.61	83.02	31.91	25.57	456.81	7.34	3.39
248.5	295.80	4720.64	79.55	26.48	25.34	446.17	7.31	2.71
252	288.88	4613.10	76.65	26.90	24.82	441.18	6.89	3.00
255.5	274.34	4495.53	75.20	27.68	23.33	409.65	6.78	2.96
259	266.51	4380.82	69.65	26.18	22.03	400.12	6.22	3.28
262.5	271.92	4299.48	75.35	27.29	23.16	390.87	6.66	3.02
266	267.41	4183.01	74.40	22.78	22.95	382.92	6.70	2.66
269.5	263.37	4102.71	65.54	25.21	22.53	374.95	5.73	2.77
273	255.36	4027.00	64.07	23.19	21.70	437.55	6.92	2.49
276.5	240.72	3903.20	68.05	21.31	20.97	357.51	5.95	2.88
280	247.00	3822.30	61.70	23.87	21.20	349.52	5.59	2.46
283.5	235.25	3718.32	59.23	24.98	20.30	341.45	5.49	2.63
287	231.48	3673.89	60.67	21.95	20.12	335.66	5.15	2.39
290.5	226.39	3572.23	60.38	24.29	19.75	326.73	5.04	2.73
294	214.74	3506.14	59.32	22.72	18.06	321.35	5.43	2.71
297.5	215.20	3420.00	59.85	20.70	18.44	313.37	5.82	2.46
301	204.62	3361.95	55.10	20.69	17.37	306.05	4.95	2.80
304.5	201.45	3280.95	53.13	19.57	17.14	298.95	4.59	2.20
308	199.07	3201.87	52.77	16.17	16.91	292.09	4.65	1.95
311.5	192.91	3143.83	49.37	17.02	16.18	287.07	4.60	2.10
315	188.64	3047.99	48.02	18.98	16.02	279.58	4.75	2.48
318.5	189.70	2968.41	46.94	18.27	16.15	272.60	4.30	2.41
322	180.42	2921.84	46.15	16.09	15.34	267.42	4.41	2.00
325.5	180.89	2847.17	48.59	16.80	15.43	260.08	4.47	2.28
329	177.25	2799.30	46.31	14.45	15.32	255.99	3.95	1.43
332.5	173.83	2705.43	45.00	15.58	14.61	285.84	5.52	1.47
336	164.85	2686.61	45.22	17.37	13.65	245.07	4.06	2.02
339.5	165.54	2588.75	40.89	16.21	14.70	237.09	3.75	1.99
343	160.17	2512.81	40.88	15.29	13.33	230.04	3.95	1.92
346.5	158.80	2485.59	41.83	6.31	13.63	226.64	3.77	1.06
350	151.62	2399.34	39.76	15.57	12.55	219.11	3.52	1.54
353.5	151.05	2376.33	39.01	11.20	12.62	217.09	3.60	1.46
357	148.09	2322.44	37.07	13.78	13.68	211.95	3.37	1.83
360.5	141.04	2273.42	38.89	13.54	11.66	206.18	3.42	1.47
364	145.13	2233.88	34.29	13.56	12.34	202.80	3.30	1.59
367.5	136.13	2187.08	36.35	10.88	11.37	198.92	3.16	1.45
371	131.61	2135.97	33.39	11.40	11.42	195.40	3.15	1.23
374.5	129.72	2077.55	34.86	12.14	11.16	190.60	3.17	1.40
378	126.37	2037.75	30.01	10.12	10.63	187.96	2.80	1.46

381.5	125.60	1955.58	33.23	12.27	11.07	179.68	3.17	1.57
385	119.57	1949.33	28.00	9.60	10.33	179.44	2.57	1.43
388.5	116.62	1886.60	30.23	12.17	10.19	173.30	3.07	1.81
392	113.91	1841.80	31.09	9.62	9.98	168.92	3.07	1.42
395.5	111.17	1821.64	26.32	9.78	9.81	167.70	2.69	1.40
399	110.44	1749.13	27.89	6.52	9.73	160.58	2.88	0.72
402.5	107.18	1726.83	30.12	10.07	9.22	158.32	2.94	0.80
406	105.16	1696.98	26.86	0.00	9.49	156.33	3.48	0.00
409.5	102.55	1640.71	23.60	10.52	8.88	150.40	2.22	1.23
413	98.57	1616.90	22.60	0.00	8.56	148.13	2.32	0.00
416.5	99.91	1575.25	24.51	8.23	8.58	144.60	2.36	0.88
420	95.64	1545.89	24.94	8.06	8.22	141.24	2.57	1.00
423.5	94.54	1505.57	5.84	8.26	8.03	137.23	1.12	0.63
427	90.61	1474.34	21.19	9.15	7.76	134.52	2.32	1.04
430.5	87.39	1434.99	22.62	0.00	7.30	131.90	2.31	0.00
434	86.85	1420.90	23.05	11.37	7.29	129.79	2.26	1.25
437.5	85.00	1376.40	19.48	0.00	7.12	126.34	1.77	0.00
441	81.60	1341.17	22.86	6.99	7.00	122.74	2.32	0.65
444.5	84.49	1332.64	18.81	0.00	7.41	121.53	1.77	0.00
448	80.35	1292.25	18.30	8.30	6.72	118.37	1.73	0.99
451.5	80.13	1251.91	18.77	0.00	6.80	115.75	1.65	0.00
455	76.37	1232.02	18.98	7.86	6.54	113.14	2.00	0.69
458.5	76.86	1201.84	20.49	0.00	6.62	110.55	2.14	0.00
462	75.65	1175.61	17.66	5.22	6.62	107.73	1.70	0.61
465.5	68.73	1138.47	14.25	0.00	6.23	104.43	1.37	0.00
469	71.83	1123.60	15.28	0.00	6.05	103.15	1.47	0.00
472.5	68.27	1100.28	16.09	0.00	5.69	118.89	1.23	0.00
476	66.99	1073.79	17.33	0.00	5.74	97.87	1.45	0.00
479.5	64.71	1045.35	15.43	0.00	5.32	95.20	1.43	0.00
483	62.76	1027.43	13.46	6.47	5.22	94.06	1.44	0.52
486.5	62.55	1001.34	13.37	0.00	5.34	90.91	1.13	0.00
490	62.86	980.82	12.81	6.48	5.05	89.89	1.34	0.66
493.5	58.49	949.34	13.12	0.00	5.04	87.38	1.01	0.00
497	55.12	929.66	15.58	5.76	4.57	85.33	1.41	0.66
500.5	55.91	909.68	13.17	0.00	4.46	83.86	1.13	0.00
504	53.75	888.72	17.82	0.00	4.69	82.45	1.87	0.00
507.5	53.59	862.47	16.49	0.00	4.68	79.95	1.41	0.00
511	52.72	844.06	12.71	8.60	4.43	78.07	1.09	1.05
514.5	54.27	821.75	12.20	0.00	4.72	87.56	1.14	0.00
518	52.08	808.65	12.90	0.00	4.23	74.86	1.01	0.00
521.5	48.58	789.14	12.05	0.00	3.89	72.40	0.90	0.00

4.2. Analysis of Cellular Metabolism with Ordinary Differential Equation (ODE) Models

To quantify the metabolic profiles from the hyperpolarized [1-^{13}C]pyruvate cellular data above, the resulting integrated signal intensity time series from the metabolites were fit to a set of ordinary differential equations (ODEs) modeling the conversion, interconversion, and decay of the metabolites. The integrals are pre-processed to exclude early-time artifacts and zero-points from the fitting protocols. The pyruvate decay was modeled as a monoexponential decay function, assuming the dominating effect of the saturated system was T_1 relaxation. A linear interpolation of this fitted decay was used as a flux source (time-varying input) of the substrate for the other metabolite downstream ODE models. In all models, a single decay term gamma (γ) was used to encompass both relaxation decay and other decay of the signal (e.g. by consumption or other metabolism of the metabolite). All models were fit using non-linear least-squares minimization via the *scipy.optimize.least_squares* solver, integrating the ODEs using *solve_ivp* over the time window of the experiment.

A reversible two compartment ODE system was used to model the interconversion of [^{13}C]bicarbonate (BIC) and $^{13}CO_2$. This model was parameterized by forward and reverse interconversion rate constants and a single decay term γ, where the same γ value was used for both BIC and $^{13}CO_2$ due to their exchange in the carbonic acid buffer system. The forward conversion of pyruvate into $^{13}CO_2$ served as the flux input into the system. To enable direct comparison with experimental data, model predictions were interpolated to experimental acquisition times, with simultaneous residual minimization across $^{13}CO_2$ and BIC profiles. The equations for this fitting are given below:

$$\frac{dCO2}{dt} = k_{PYR\to CO2}PYR + k_{BC\to CO2}BIC - k_{CO2\to BC}CO2 - CO2\gamma_{CO2/BIC} \quad \textbf{Eq. S4}$$

$$\frac{dBIC}{dt} = k_{CO\to BC}CO2 - k_{BC\to CO}BIC - BIC\gamma_{CO2/BIC} \quad \textbf{Eq. S5}$$

To assess the accumulation of [1-^{13}C]pyruvate hydrat, each signal was fit using a single-compartment accumulation model, in which metabolite production from pyruvate (via a fitted rate constant k) was balanced by a first-order decay term (γ). The equations for this fitting is provided below, where *M* represents either [1-^{13}C]pyruvate hydrate.

$$\frac{dM}{dt} = k_M PYR - M\gamma_M \quad \textbf{Eq. S6}$$

The code for this metabolite fitting protocol is separately provided.

The fits for the de-noised and integrated data from samples C1, C2, and C3 are shown (**Table S8, S9, S10**) are shown below (**Figures S8, S9 and S10**)C1, is shown in **Figure 4** of the main text.

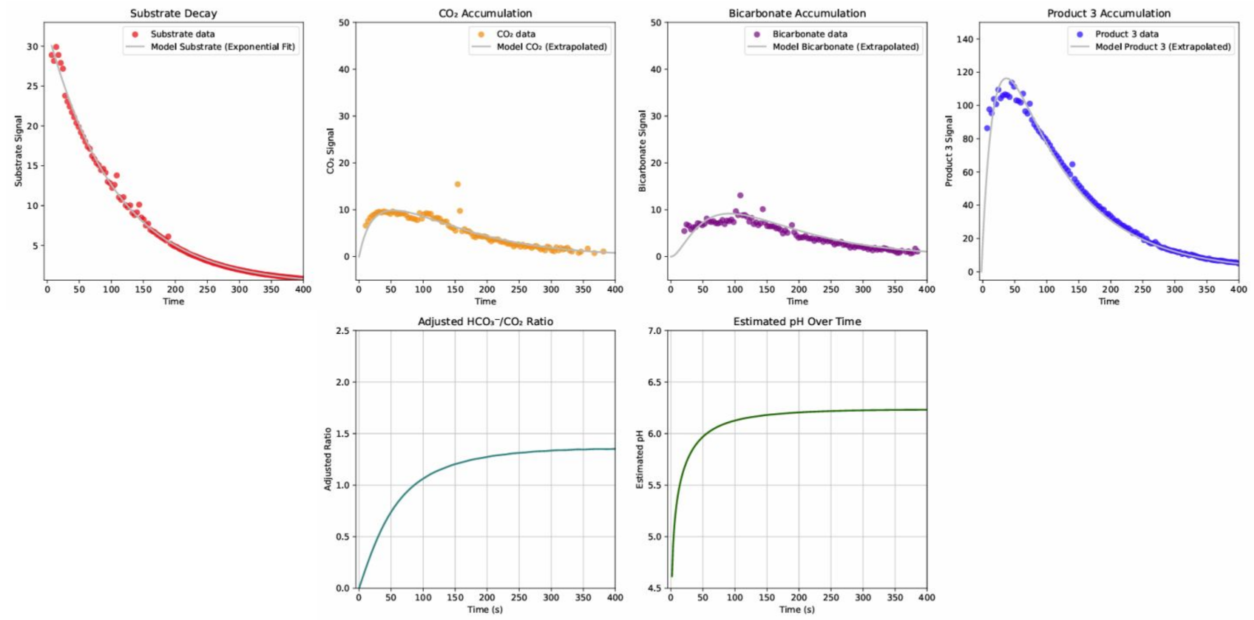

Figure S8. Sample C1 fits for pyruvate and the pyruvate hydrate, bicarbonate, and CO_2 products using the protocol described above. This data is also shown in Figure 4 of the main text.

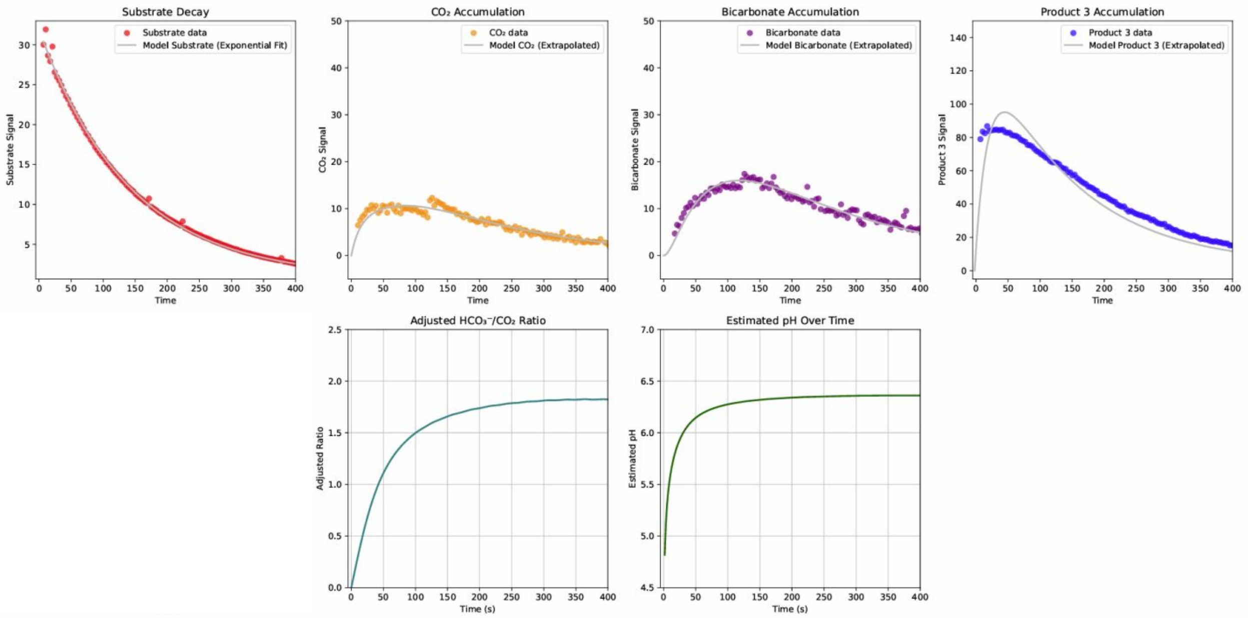

Figure S9. Sample C2 fits for pyruvate and the pyruvate hydrate, bicarbonate, and CO_2 products using the protocol described above.

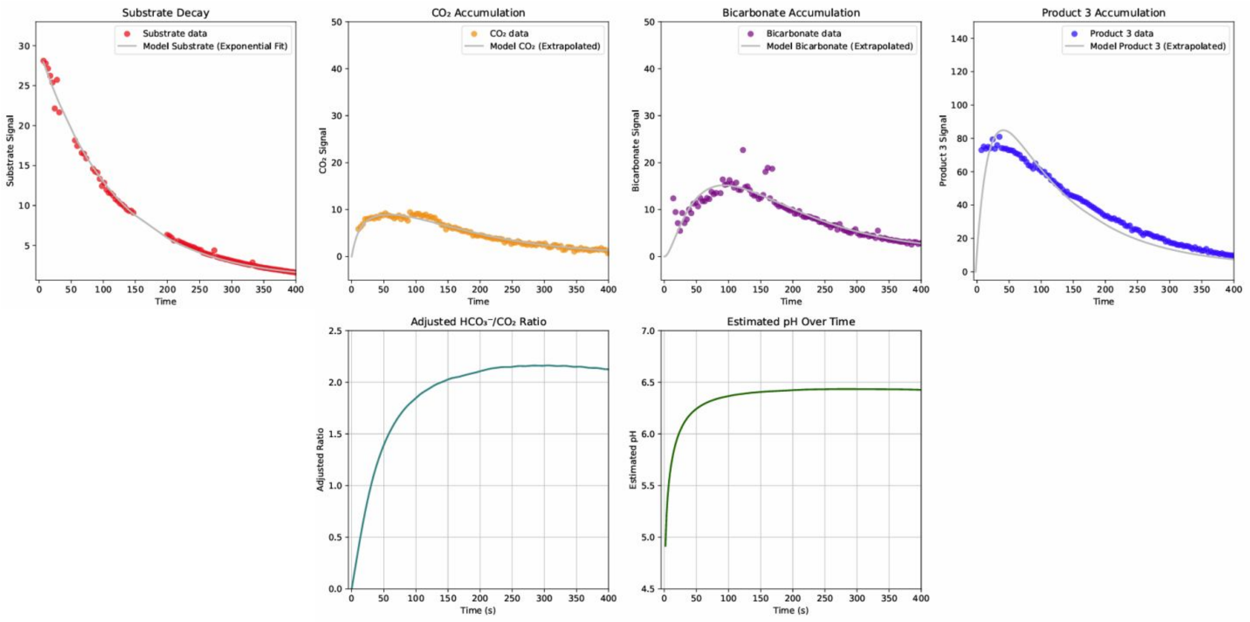

Figure S10. Sample C3 fits for pyruvate and the pyruvate hydrate, bicarbonate, and CO_2 products using the protocol described above.

In the main text, we present the average of the fitting parameters for all three data sets in **Table 2**. Given below in **Table S11** are the individual parameters for each data set.

Table S11. Individual sample metabolite profile fitting parameters (Sample C1, C2, and C3)

Sample #	tau_sub1	ksubCO	kBCCO	kCOBC	gamCO/BC	kPH	gamPH
Sample C1	107.02	0.00021	0.03772	0.01871	0.01797	0.002803	0.05474
Sample C2	140.58	0.00021	0.05992	0.02569	0.01252	0.001992	0.049122
Sample C3	117.1	0.00027	0.07485	0.02468	0.01662	0.002048	0.051053

4.3. Intracellular pH Estimation using Metabolite Fitting

Dynamic pH values were determined over time based on the ratio of modeled bicarbonate to CO_2 concentrations generated from the two-compartment kinetic model. At each time point, the instantaneous BIC/CO_2 ratio was calculated from the ODE-derived metabolite concentrations and converted to pH using the modified Henderson-Hasselbalch equation (**Eq. S7**), assuming a pKa of 6.1 for the carbonic acid buffering system.[13]

$$pH = pK_a + \log_{10}\left(\frac{HCO_3^-}{CO_2}\right) \qquad \textbf{Eq. S7}$$

The resulting pH trace provides a dynamic view of the intracellular environment as it evolves throughout the course of the experiment, capturing transient shifts in pH that arise from the ongoing enzymatic and transport-mediated interconversion between CO_2 and bicarbonate. These deviations from equilibrium reflect the influence of metabolic activity, compartmental transport,

and buffering capacity. To distill this time-resolved behavior into a representative summary value, the instantaneous BIC/CO_2 ratios (derived from the ODE-modeled metabolite concentrations) were used to compute an average over the full experimental time window. This ratio was then converted to an average pH value using the modified Henderson-Hasselbalch equation.

5. Supporting Information References

(1) Adams, R. W.; Aguilar, J. A.; Atkinson, K. D.; Cowley, M. J.; Elliott, P. I. P.; Duckett, S. B.; Green, G. G. R.; Khazal, I. G.; López-Serrano, J.; Williamson, D. C. Reversible Interactions with Para-Hydrogen Enhance NMR Sensitivity by Polarization Transfer. *Science* **2009**, *323* (5922), 1708–1711.

(2) Tickner, B. J.; Semenova, O.; Iali, W.; Rayner, P. J.; Whitwood, A. C.; Duckett, S. B. Optimisation of Pyruvate Hyperpolarisation Using SABRE by Tuning the Active Magnetisation Transfer Catalyst. *Catal. Sci. Technol.* **2020**, *10* (5), 1343–1355.

(3) Nantogma, S.; Joalland, B.; Wilkens, K.; Chekmenev, E. Y. Clinical-Scale Production of Nearly Pure (>98.5%) Parahydrogen and Quantification by Benchtop NMR Spectroscopy. *Anal. Chem.* **2021**, *93* (7), 3594–3601.

(4) TomHon, P.; Abdulmojeed, M.; Adelabu, I.; Nantogma, S.; Kabir, M. S. H.; Lehmkuhl, S.; Chekmenev, E. Y.; Theis, T. Temperature Cycling Enables Efficient 13C SABRE-SHEATH Hyperpolarization and Imaging of [1-13C]-Pyruvate. *J. Am. Chem. Soc.* **2022**, *144* (1), 282–287.

(5) McBride, S. J.; Pike, M.; Curran, E.; Zavriyev, A.; Adebesin, B.; Tucker, L.; Harzan, J. M.; Senanayake, I. M.; Shen, S.; Abdulmojeed, M.; Theiss, F.; Boele, T.; Gade, T. P.; Duckett, S.; Goodson, B. M.; Rosen, M. S.; Chekmenev, E. Y.; Yuan, H.; Dedesma, C.; Kadlecek, S.; Theis, T.; TomHon, P. Scalable Hyperpolarized MRI Enabled by Ace-SABRE of [1-13C]Pyruvate. *Angew. Chem. Int. Ed.* **2025**, *n/a* (n/a), e202501231.

(6) Nantogma, S.; Chowdhury, M. R. H.; Kabir, M. S. H.; Adelabu, I.; Joshi, S. M.; Samoilenko, A.; de Maissin, H.; Schmidt, A. B.; Nikolaou, P.; Chekmenev, Y. A.; Salnikov, O. G.; Chukanov, N. V.; Koptyug, I. V.; Goodson, B. M.; Chekmenev, E. Y. MATRESHCA: Microtesla Apparatus for Transfer of Resonance Enhancement of Spin Hyperpolarization via Chemical Exchange and Addition. *Anal. Chem.* **2024**, *96* (10), 4171–4179.

(7) Waddell, K. W.; Coffey, A. M.; Chekmenev, E. Y. In Situ Detection of PHIP at 48 mT: Demonstration Using a Centrally Controlled Polarizer. *J. Am. Chem. Soc.* **2011**, *133* (1), 97–101.

(8) Verduyn, C.; Stouthamer, A. H.; Scheffers, W. A.; van Dijken, J. P. A Theoretical Evaluation of Growth Yields of Yeasts. *Antonie Van Leeuwenhoek* **1991**, *59* (1), 49–63.

(9) Vaziri, S.; Autry, A. W.; Lafontaine, M.; Kim, Y.; Gordon, J. W.; Chen, H.-Y.; Hu, J. Y.; Lupo, J. M.; Chang, S. M.; Clarke, J. L.; Villanueva-Meyer, J. E.; Bush, N. A. O.; Xu, D.; Larson, P. E. Z.; Vigneron, D. B.; Li, Y. Assessment of Higher-Order Singular Value Decomposition Denoising Methods on Dynamic Hyperpolarized [1-13C]Pyruvate MRI Data from Patients with Glioma. *NeuroImage Clin.* **2022**, *36*, 103155.

(10) Kim, Y.; Chen, H.-Y.; Autry, A. W.; Villanueva-Meyer, J.; Chang, S. M.; Li, Y.; Larson, P. E. Z.; Brender, J. R.; Krishna, M. C.; Xu, D.; Vigneron, D. B.; Gordon, J. W. Denoising of Hyperpolarized 13 C MR Images of the Human Brain Using Patch-Based Higher-Order Singular Value Decomposition. *Magn. Reson. Med.* **2021**, *86* (5), 2497–2511.

(11) Larson, P. E. Z.; Bernard, J. M. L.; Bankson, J. A.; Bøgh, N.; Bok, R. A.; Chen, A. P.; Cunningham, C. H.; Gordon, J. W.; Hövener, J.-B.; Laustsen, C.; Mayer, D.; McLean, M. A.; Schilling, F.; Slater, J. B.; Vanderheyden, J.-L.; von Morze, C.; Vigneron, D. B.; Xu, D.

Current Methods for Hyperpolarized [1-13C]Pyruvate MRI Human Studies. *Magn. Reson. Med.* **2024**, *91* (6), 2204–2228.
(12) Koehl, P. Linear Prediction Spectral Analysis of NMR Data. *Prog. Nucl. Magn. Reson. Spectrosc.* **1999**, *34* (3), 257–299.
(13) Shaw, I.; Gregory, K. Acid–Base Balance: A Review of Normal Physiology. *BJA Educ.* **2022**, *22* (10), 396–401.
